# Higher Fecal Short-Chain Fatty Acid Levels Are Associated with Gut Microbiome Dysbiosis, Obesity, Hypertension and Cardiometabolic Disease Risk Factors

**DOI:** 10.3390/nu11010051

**Published:** 2018-12-27

**Authors:** Jacobo de la Cuesta-Zuluaga, Noel T. Mueller, Rafael Álvarez-Quintero, Eliana P. Velásquez-Mejía, Jelver A. Sierra, Vanessa Corrales-Agudelo, Jenny A. Carmona, José M. Abad, Juan S. Escobar

**Affiliations:** 1 Vidarium–Nutrition, Health and Wellness Research Center, Grupo Empresarial Nutresa, Calle 8 sur #50-67, 050023 Medellin, Colombia; jdelacuesta@tuebingen.mpg.de (J.d.l.C.-Z.); epvelasquez@serviciosnutresa.com (E.P.V.-M.); jasierra@serviciosnutresa.com (J.A.S.); vcorrales@serviciosnutresa.com (V.C.-A.); 2 Department of Epidemiology, Johns Hopkins Bloomberg School of Public Health, 615 N. Wolfe Street, Baltimore, MD 21205, USA; noeltmueller@jhu.edu; 3 Welch Center for Epidemiology, Prevention and Clinical Research, Johns Hopkins Medical Institutions, 2024 E. Monument Street, Baltimore, MD 21205, USA; 4 Grupo de Investigación en Sustancias Bioactivas, Sede de Investigación Universitaria (SIU), Universidad de Antioquia, Calle 62 #52-59, 050010 Medellin, Colombia; malvarez153@gmail.com; 5 Dinámica IPS, Especialista en Ayudas Diagnósticas, Calle 27 #45-109, 050021 Medellin, Colombia; jenncava@dinamicaips.com.co; 6 EPS SURA, Calle 49A #63-55, 050034 Medellin, Colombia; jabad@sura.com.co

**Keywords:** gut microbiota, SCFA, butyrate, adiposity, metabolic dysregulation, hypertension, gut permeability

## Abstract

Fiber fermentation by gut microbiota yields short-chain fatty acids (SCFAs) that are either absorbed by the gut or excreted in feces. Studies are conflicting as to whether SCFAs are beneficial or detrimental to cardiometabolic health, and how gut microbiota associated with SCFAs is unclear. In this study of 441 community-dwelling adults, we examined associations of fecal SCFAs, gut microbiota diversity and composition, gut permeability, and cardiometabolic outcomes, including obesity and hypertension. We assessed fecal microbiota by 16S rRNA gene sequencing, and SCFA concentrations by gas chromatography/mass spectrometry. Fecal SCFA concentrations were inversely associated with microbiota diversity, and 70 unique microbial taxa were differentially associated with at least one SCFA (acetate, butyrate or propionate). Higher SCFA concentrations were associated with a measure of gut permeability, markers of metabolic dysregulation, obesity and hypertension. Microbial diversity showed association with these outcomes in the opposite direction. Associations were significant after adjusting for measured confounders. In conclusion, higher SCFA excretion was associated with evidence of gut dysbiosis, gut permeability, excess adiposity, and cardiometabolic risk factors. Studies assessing both fecal and circulating SCFAs are needed to test the hypothesis that the association of higher fecal SCFAs with obesity and cardiometabolic dysregulation is due to less efficient SCFA absorption.

## 1. Introduction

One of the main functions of the human intestinal microbiota is to ferment indigestible dietary fiber in the large intestine. The products of this fermentation process are short-chain fatty acids (SCFAs), including acetate, propionate, and butyrate [1]. SCFAs can either be excreted in the feces or taken up by the gut epithelium to participate in a variety of physiologic processes. Uptake of SCFAs involves poorly selective anion-transporting proteins expressed by the gut epithelium, whose purpose is to maximize the amount of SCFAs absorbed from the lumen [2]. Butyrate, in particular, serves as the primary energy source for colonocytes [3]. Other molecules enter the portal circulation where they can be metabolized by the liver, or released into the systemic circulation where they can bind to SCFA receptors in the vascular epithelium or afferent arterioles and alter cardiometabolic health [4].

Several lines of evidence suggest that SCFAs may be beneficial for cardiometabolic health. In vitro experiments have demonstrated that butyrate plays a key role in the maintenance of gut-barrier function by preserving luminal anaerobiosis through the stabilization of specific transcription factors, assembly of tight junction proteins, and mucin secretion [5,6,7]. By strengthening the intestinal barrier, butyrate may block the translocation of lipopolysaccharide (LPS)—a potent inflammatory molecule produced in the cell membrane of Gram-negative bacteria—which has been associated with metabolic endotoxemia, inflammation, insulin resistance, adiposity, and hepatic fat [8]. SCFAs may also be involved in appetite regulation [9], and play a role in gut and peripheral immune responses [10]; inducing the differentiation of T-regulatory cells [11].

Yet not all evidence supports a beneficial role for SCFAs. An estimated 5–10% of daily calories come from the oxidation of SCFAs, and their metabolites can be utilized for de novo lipid and glucose synthesis [12]. Correspondingly, murine models and epidemiologic studies have consistently shown that higher fecal SCFA concentrations are positively associated with body weight [13,14,15], and increase with calorie-rich diets [16]. Conversely, lower fecal SCFA levels have been associated with leanness [15]. Moreover, metagenomic studies of obesity have shown that the obese human gut microbiome is enriched for pathways involved in microbial processing of carbohydrates (e.g., phosphotransferase systems), as well as in genes related to SCFA production (e.g., acetyl/propionyl-CoA carboxylase and acetyl-CoA carboxylase) [17].

In summary, in vitro experiments have demonstrated beneficial physiologic effects of SCFAs, but human studies suggest that fecal SCFA concentrations are associated with excess adiposity. Given this incongruent evidence, we aimed to examine associations of fecal microbiota and SCFA concentrations with gut permeability, adiposity, and cardiometabolic health outcomes in a community-based sample of male and female adults.

## 2. Materials and Methods

### 2.1. Study Population

We enrolled 441 men and women, 18 to 62 years of age, living in the cities of Bogota, Medellin, Cali, Barranquilla, and Bucaramanga (Colombia, South America) between July and November 2014. Participants were enrolled in similar proportions according to: Category of body mass index (BMI: Normal weight, overweight, and obese); city of residence; sex; and age range (18–40 and 41–62 years). We excluded underweight participants (i.e., BMI < 18.5 kg/m^2^), pregnant women, individuals who had consumed antibiotics or antiparasitics in the three months prior to enrollment, and individuals diagnosed with neurodegenerative diseases, gastrointestinal diseases (Crohn’s disease, ulcerative colitis, short bowel syndrome, diverticulosis or celiac disease), or cancer within the year before enrollment.

This study followed the principles of the Declaration of Helsinki and had minimal risk according to the Colombian Ministry of Health (Resolution 8430 of 1993). Written informed consent was obtained from all the participants prior to the beginning of the study. This study was approved by the Bioethics Committee of SIU—Universidad de Antioquia (act 14-24-588 dated 28 May 2014).

### 2.2. Blood Biochemical Parameters

We collected fasting peripheral venous blood and isolated the serum by centrifugation. High density lipoprotein (HDL) cholesterol, low density lipoprotein (LDL) cholesterol, very low density lipoprotein (VLDL) cholesterol, triglycerides, and fasting glucose were measured by colorimetric enzymatic assays (cobas 701, Roche, Mannheim, Germany); fasting insulin by a chemiluminescence immunoassay (cobas E411); glycated hemoglobin (HbA1c) by high-performance liquid chromatography (Premier Hb9210, Labcare de Colombia, Cota, Colombia); leptin by micro ELISA (DSX-ELISA Processing System, Dynex, Louvain-la-Neuve, Belgium); adiponectin by the lanthanide chelate excite ultra assay (Perkin Elmer, Waltham, MA, USA); and high-sensitivity C-reactive protein (hs-CRP) by a particle-enhanced immunoturbidimetric assay (cobas 502). Fasting blood insulin and glucose were used to calculate the insulin resistance index using the homeostasis model assessment (HOMA-IR).

Concentrations of serum lipopolysaccharide-binding protein (LBP), a biomarker produced in response to LPS microbial translocation and a marker of gut permeability [18], were measured in duplicate in samples diluted 1:1000, using an ELISA kit (Duoset, R&D Systems, Minneapolis, MN, USA) per the manufacturer’s instructions. The absorbance values for the ELISA were determined using a Synergy HT Microplate Reader (Bio-Tek Instruments, Winooski, VT, USA) at an optical absorbance of 450 nm, corrected at 570 nm. Final concentrations were determined with reference to a standard curve.

### 2.3. Adiposity and Blood Pressure

Weight, height, waist circumference and four skinfold thicknesses (biceps, triceps, subscapular, and ileocrestal) were measured with internationally recognized techniques after training and standardizing the evaluators. Weight was measured with Cardinal Detecto DR400C digital scales (Webb City, MO, USA), and height with the Seca portable measuring rods (Hamburg, Germany). We calculated BMI as weight (kg)/height squared (m^2^). Waist circumference was measured with Mabis measuring tapes (Waukegan, IL, USA) and skinfolds with the Guide Slim adipometers (Plymouth, MI, USA). Skinfold measurements were used to calculate body fat percentage—the logarithm of the sum of the four folds allowed for a calculation of body density—which was used to estimate the body fat percentage using a validated equation [19]. Blood pressure was measured using a Rossmax AF701f digital blood pressure monitor (Berneck, Switzerland), and recorded systolic (SBP) and diastolic (DBP) blood pressures in mm Hg. Mean arterial pressure (MAP) was calculated as (2 × DBP + SBP)/3. All equipment was calibrated at the beginning of the study.

### 2.4. Diet Assessment and Physical Activity

We trained research staff to carry out 24-h dietary recall interviews in a standardized fashion. They captured detailed information about all foods and beverages consumed by the respondent in the past 24 h. Interviews were randomly distributed in the different days of the week. Interviewers used validated forms, food models, geometric figures, and full-size pictures to assess portion sizes and improve accuracy. Ten percent of the participants were interviewed a second time on a different day of the week, with a minimum of two days between consecutive evaluations, to estimate intra-individual variability. Estimations of total energy intake and dietary fiber were obtained for each participant using the EVINDI 4.0 and PC-SIDE 1.0 software.

Physical activity was assessed using the International Physical Activity Questionnaire-short form (IPAQ-SF), Colombian Spanish version adapted 4/2003. The number of metabolic equivalents (MET) per units of time (MET/min/week) was quantified according to the IPAQ guidelines [20].

### 2.5. Fecal Sampling

Participants collected fecal samples in two hermetically-sealed, sterile receptacles provided by the research team. Samples were immediately refrigerated in household freezers and brought to a collection center within 12 h. The first sample served to evaluate stool consistency (diarrheic, mushy, normal, or hard) by trained laboratory technicians; the second sample was stored on dry ice and sent to a central laboratory via next-day delivery. Upon receipt, samples were aliquoted and kept at −80 °C until further analysis.

### 2.6. Fecal Microbiota Characterization

A detailed description of the laboratory and bioinformatic procedures we used to generate, process, and analyze the fecal microbiome of our study participants can be found elsewhere [21]. Briefly, microbial DNA was extracted from the fecal aliquots using the QIAamp DNA Stool Mini Kit (Qiagen; Hilden, Germany). The V4 region of the 16S rRNA gene was amplified with the primers F515 and R806, and sequenced with the Illumina MiSeq v2 platform. To examine the influence of reagent contamination, a negative control (ultrapure water), a DNA extraction blank, and a mock community (HM-782D, BEI Resources, Manassas, VA, USA) were included in the analyses. In addition, we randomized the sequencing order. Likewise, we assessed the reproducibility between sequencing runs by including replicate samples and determined their differences in operational taxonomic units (OTU) counts.

Amplicons were processed using Mothur v.1.36 [22] following its standard operating procedure available November 2015. OTUs delimited at 97% identity were generated and classified using Greengenes 13_8_99 [23]. The number of sequences per individual ranged from 3667 to 102,700, with a median sequence count of 28,561. A total of 4720 unique OTUs were observed, with a Matthew’s correlation of 0.79, indicating high quality clustering. Parallel sequencing of a mock community revealed a mean sequencing error rate of 0.12%, and sequencing of replicate samples in different runs indicated that the difference between sequencing runs was negligible (maximum sequence count difference between OTUs of replicate samples on rarefied data for all replicates = 85 reads; overall median differences = 0 reads).

Estimates of intra- and inter-subject diversities (alpha and beta diversities, respectively) were obtained with BiodiversityR [24] and GUniFrac [25]. The observed OTU richness was used as the primary estimate of alpha diversity. Weighted and unweighted UniFrac distances were used as estimates of beta diversity. Diversity metrics were obtained on sequence counts rarefied to 3667 sequences per sample.

### 2.7. Quantification of Fecal SCFAs

We first calibrated our fecal SCFA quantification method using a pool of five fecal samples. Fecal samples were homogenized and diluted with distilled-deionized water in a ratio 1:1. An aliquot of 1 g was spiked with a combined standard solution of SCFAs diluted in water (organic acid kit ref. 47264, Supelco (Bellefonte, PA): Acetic acid—ref. R475165; propionic acid—ref. R412368; butyric acid—ref. R420040; isobutyric acid—ref. R412415) to obtain curves in the range 25–750 ng/mL (6 concentrations; 9 replicates). We prepared the standard solution of SCFAs on the day of analysis. Linearity (homoscedasticity test, analysis of residuals), precision (RSD 3.0 for the detection of each analyte), and accuracy (3-way test of concentration for each analyte and 3 replicates; value = G_table_ (a = 0.05; k = 3; n = 3) = 0.871 (G_exp_ < G_table_ acceptance)) were considered in the evaluation of the analytic method.

We next quantified SCFAs in all fecal samples. For this, approximately 1 g of each sample was weighed into a vial of 20 mL with a magnetic thread cap and PTFE septum. The volatiles were sampled using a CTC Combipal 3 autosampler in HS/SPME mode equipped with a gray fiber (Carboxen/DVB/PDMS—ref. SU57329U, Supelco). The autosampler was programmed as follows: Fiber-conditioning module, 5 min at 250 °C; sample equilibration, 30 min at 80 °C; extraction of analytes, 25 min at 80 °C; desorption of the fiber, 1 min at 250 °C. The analytes were injected in the splitless mode into an Agilent 7890 gas chromatography (GC) system (Wilmington, DE, USA) equipped with a 5975C mass spectrometer (MS) detector and an Agilent J & W DB-WAX column (30 m, 0.25 mm, 0.250 µm). The oven temperature was programmed to start at 80 °C (5 min), to increase to 100 °C for 1 min at 3 °C/min, and then to increase to 250 °C for 1 min at 6 °C/min. The MS was tuned during all experiments; the signal acquisition for quantification was done in the single-ion monitoring (SIM) mode (Table S1). The temperature of the ionization source and the quadrupole were 230 °C and 150 °C, respectively. The electron-impact ionization energy was 70 eV. The chromatographic peaks were checked for homogeneity using the extracted ions of the characteristic fragments to optimize the resolution and peak symmetry. We performed data analysis using a MassHunter WorkStation (Agilent; Santa Clara, CA, USA). Concentrations of acetate, propionate, butyrate, and isobutyrate were expressed in µmol/g of feces. Total SCFAs were the sum of acetate, propionate, and butyrate.

### 2.8. Statistical Analyses

Our primary analyses were conducted among all participants (*N* = 441). Non-normally distributed variables were transformed using natural logarithm for continuous variables and arcsine-square-root for proportions. For appropriate logarithmic transformation, it was necessary to add constants to some variables to avoid infinite values (1 to physical activity; 0.5 to adiponectin; 0.1 to total SCFAs and acetate; 0.05 to propionate; 0.01 to butyrate; and 0.001 to isobutyrate).

To understand how the participant characteristics varied across levels of the dependent variables in our study, we calculated and presented unadjusted means, or percentages when appropriate, of demographic, lifestyle and clinical factors across tertiles of fecal butyrate concentrations and gut microbiota diversity. Tertile 1 corresponds to the lowest level, tertile 2 the intermediate level, and tertile 3 the highest level. We tested for significant trends across tertiles using ANOVA (for continuous variables) or chi-squared tests (for categorical variables), and adjusted *p*-values for multiple comparisons [26]. Similar comparisons were performed across tertiles of acetate, propionate, isobutyrate, and total SCFAs.

To address potential confounding associations, we next performed various regression models adjusted for pertinent covariates. Our main multivariable models included participant age (years; continuous), city of residence (Bogota, Medellin, Cali, Barranquilla and Bucaramanga), physical activity (MET/min/week; continuous), fiber intake (g/day; continuous), and total caloric intake (kcal/day; continuous). We adjusted for dietary fiber intake because it is the main substrate for SCFA production, and it is associated with obesity and cardiometabolic risk factors. Similarly, adjustment for caloric intake was justified because individuals might have higher SCFA levels because they eat more overall, which can contribute to obesity. The other covariates included in multivariable models also constitute potential confounders because of their association with cardiometabolic health, microbiota diversity, and SCFA production [27,28,29].

After evaluating non-linear associations with restricted cubic splines, we proceeded to use multivariable-adjusted models to examine differences in microbiota diversity and cardiometabolic parameters by tertiles of SCFAs. Procrustes analysis [30] was implemented with 10,000 permutations to explore associations of microbial community beta diversity with multivariable-adjusted SCFA concentrations (total SCFAs, acetate, propionate, butyrate, and isobutyrate), BMI, waist circumference, and blood pressure. Multivariable-adjusted Pearson correlation coefficients were calculated between pairs of variables (SCFAs, adiposity, blood chemistry, blood pressure, and microbiota OTU richness), and adjusted *p*-values were obtained using the Benjamini and Hochberg correction (FDR).

To determine associations between specific microbial taxa and SCFAs, we first extracted OTUs with median abundances ≥0.001% and then fitted the quasi-Poisson generalized linear models on rarefied sequence counts. Multivariable-adjusted Spearman correlation coefficients between OTU abundances and individual SCFAs were next calculated, and FDR-adjusted *p*-values were obtained [26]. Finally, we used log-binomial multivariable regression models with robust variance to examine tertiles of butyrate (and the other SCFAs) and microbiota diversity in relation to the prevalence of obesity (defined as BMI ≥ 30 kg/m^2^), central obesity (defined as waist circumference ≥102 cm in men, or ≥88 cm in women), and hypertension (defined as SBP ≥ 130 mm Hg, or DBP ≥ 80 mm Hg, or previous diagnosis of hypertension or use of antihypertensive medications). Log-binomial multivariable regression models were restricted to individuals with no missing values (*N* = 431).

To determine the robustness of our findings, we conducted sensitivity analyses in the subset of participants that did not report smoking or use of pharmacological treatments (with the exception of over-the-counter vitamin and mineral supplements, phytotherapeutics, and contraceptives) in self-administered questionnaires (*N* = 217). This excluded participants taking drugs known to affect SCFAs, such as metformin [21,31]. Finally, to determine if SCFA excretion was associated with transit time, we examined whether adjusted fecal SCFA levels were higher in individuals with diarrheic stools than those with normal or solid stools.

### 2.9. Availability of Data and Material

Raw 16S rRNA gene reads were deposited at the short read archive (BioProject PRJNA417579). The employed code for statistical analyses is available at Github (https://github.com/jsescobar/scfa). The parameters related to the participants’ health, diet, physical activity, and SCFAs analyzed during the current study are available from the corresponding author on reasonable request.

## 3. Results

For the 212 males and 229 females enrolled in our study, we found that participants with higher fecal butyrate excretion were more likely to be male (*q* = 0.03), to consume more dietary fiber (*q* = 0.03) and total calories (*q* = 0.05), and to have higher overall and central obesity, hypertension and, more generally, metabolic dysregulation. The latter included higher BMI (*q* = 0.03), percentage body fat (*q* = 0.04), waist circumference (*q* = 0.03), VLDL cholesterol (*q* = 0.05), triglycerides (*q* = 0.05), hs-CRP (*q* = 0.03), insulin (*q* = 0.03), and blood pressure (*q* = 0.03) among participants with higher fecal butyrate levels. Higher fecal butyrate was also associated with lower gut microbiota diversity (i.e., OTU richness; *q* = 0.03; Table 1). Trends for other SCFAs were similar (Table S2). Participants with higher gut microbiota diversity were more likely to be female (*q* = 0.04), and on average they had lower BMI (*q* = 0.04), waist circumference (*q* = 0.04), VLDL cholesterol (*q* = 0.04), triglycerides (*q* = 0.04), hs-CRP (*q* = 0.04), LBP (*q* = 0.04), and blood pressure (*q* = 0.04), while having *higher* HDL cholesterol (*q* = 0.04) and adiponectin (*q* = 0.04; Table 1).

**Figure 1:**
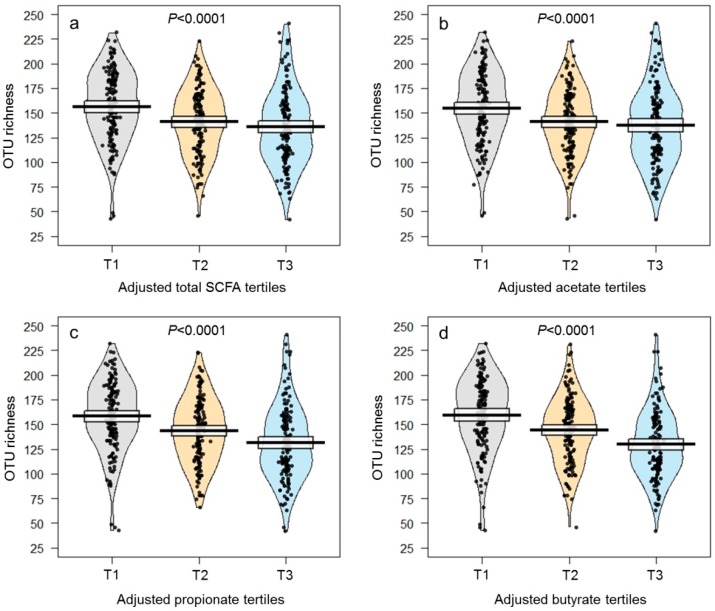
Distribution of gut microbiota diversity according to tertiles (T1: Low, T2: Intermediate, T3: High) of multivariable-adjusted fecal SCFA concentrations: (**a**) Total SCFAs; (**b**) acetate; (**c**) propionate; and (**d**) butyrate. SCFA concentrations were adjusted for age, city of origin, caloric intake, physical activity, and fiber intake. The raw data, average, and 95% confidence intervals are shown for each tertile.

**Figure 2:**
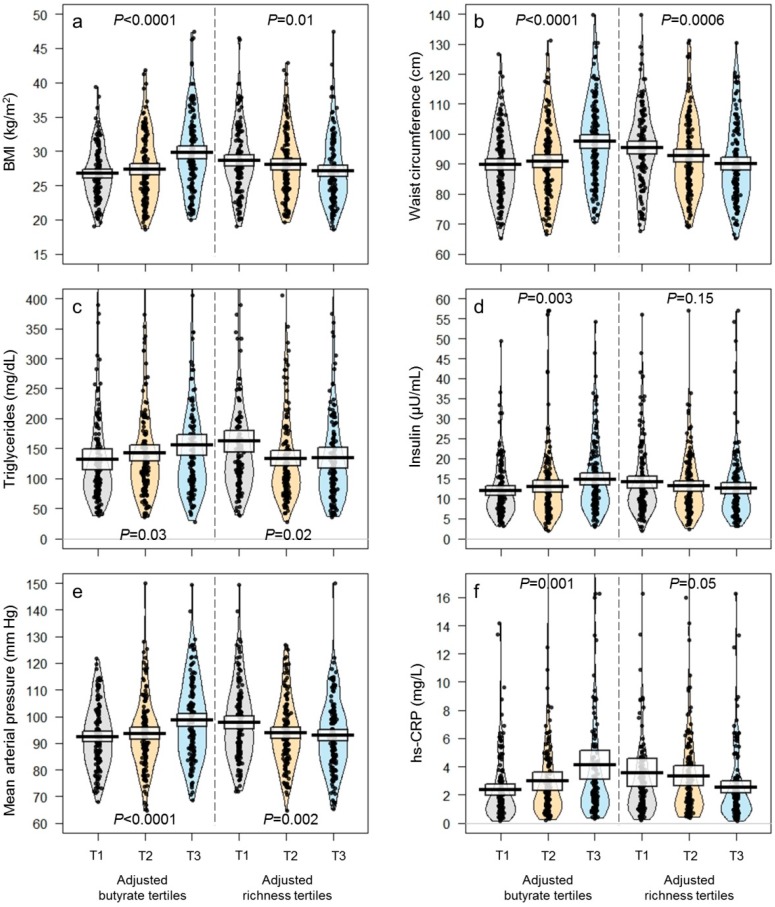
Distribution of various cardiometabolic health indicators according to tertiles (T1: Low, T2: Intermediate, T3: High) of multivariable-adjusted fecal butyrate concentrations (left) and microbiota diversity (right). (**a**) BMI; (**b**) waist circumference; (**c**) triglycerides; (**d**) fasting insulin; (**e**) blood pressure; and (**f**) hs-CRP. Values were adjusted for age, city of origin, caloric intake, physical activity, and fiber intake. The raw data, mean, and 95% confidence intervals are shown for each tertile.

Next, we found that the inverse association between fecal butyrate (and other SCFAs) and gut microbiota diversity persisted after adjustment for confounders (*q* < 0.0001; Figure 1, Table 2, and Figure S1). High microbiota diversity was also significantly associated with less gut permeability (i.e., lower LBP levels; *q* = 0.019), less adiposity (i.e., lower BMI and waist circumference; *q* < 0.05), and an overall improved cardiometabolic health profile, after multivariable adjustment (*q* < 0.05; Table 2). Interestingly, fecal SCFAs showed exactly the opposite pattern of association with adiposity and cardiometabolic indicators (*q* < 0.05; Figure 2, Table 2, Figures S2 and S3). Further, fecal SCFA levels were higher in individuals with diarrheic stools and lower in individuals with normal or solid stools (acetate: *p* = 0.06, propionate: *p* = 0.0003, butyrate: *p* < 0.0001, total SCFAs: *p* = 0.005), suggesting intestinal transit time is associated with fecal SCFA excretion (Table S3).

Using UniFrac-based beta-diversity analyses, we then demonstrated that the phylogenetic composition of the participants’ gut microbial communities was associated with multivariable-adjusted fecal SCFA concentrations (Figure S4) and adjusted indicators of cardiometabolic health (BMI, waist circumference, and blood pressure; *q* < 0.005 in Procrustes analysis with the weighted UniFrac distances for all outcomes; Table S4). In other words, fecal excretion of SCFAs and cardiometabolic disease indicators were associated with overall gut microbiota community composition.

**Figure 3:**
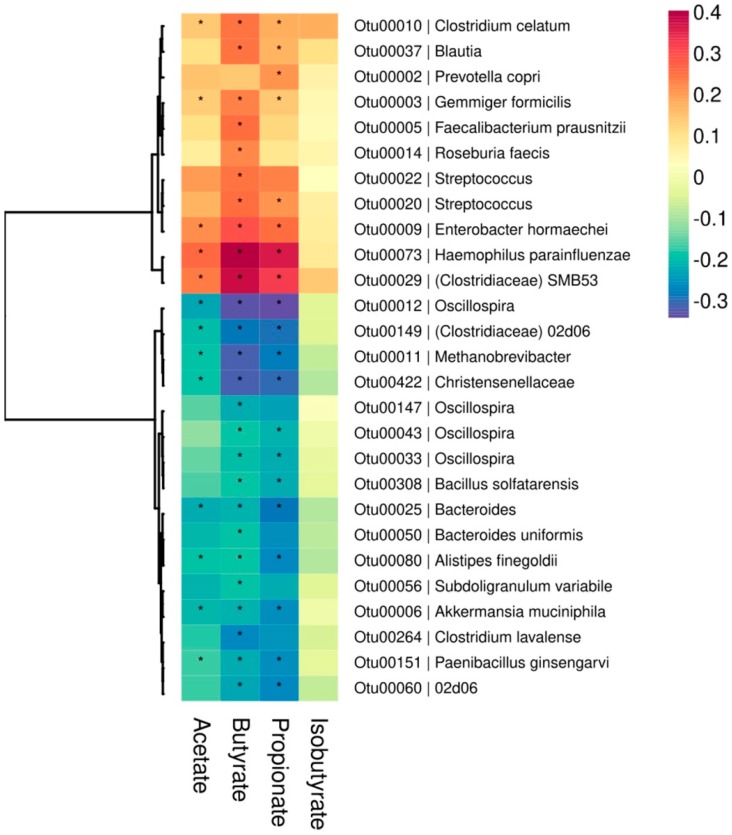
Heatmap showing the correlations between rarefied OTU abundances and multivariable-adjusted fecal SCFA concentrations. OTUs with moderate or strong association with at least one of the measured SCFAs are shown (|rho| > 0.2). The dendrogram to the left was obtained by hierarchical Ward-linkage clustering based on correlation coefficients of the relative abundances of the OTUs that had median abundances ≥0.001%. Models were adjusted for age, city of origin, caloric intake, physical activity, and fiber intake. The color scale indicates the Spearman’s correlation coefficients. FDR-adjusted *p*-values from quasi-Poisson generalized linear models are indicated (* =*q* < 0.10).

To identify the microbial taxa responsible for the differences in overall gut microbiota composition, we fitted multivariable-adjusted quasi-Poisson generalized linear models and found that 70 OTUs were significantly associated with at least one of the fecal SCFAs evaluated in our study (*q* < 0.10). Of these, 27 showed moderate-to-strong correlations (|rho| > 0.20; Figure 3). Higher butyrate excretion was positively associated with the abundances of known butyrate-producers, including *Faecalibacterium prausnitzii*, *Roseburia faecis*, and other *Clostridiales*. Other microbial taxa not known for their fermentation capacity were also positively associated with excretion of butyrate and other SCFAs. These included *Enterobacter hormaechei*, *Haemophilus parainfluenzae*, and *Streptococcus*. In contrast, the relative abundances of *Akkermansia muciniphila*, *Alistipes finegoldii*, *Bacteroides*, *Christensenellaceae*, *Methanobrevibacter*, and *Oscillospira*, among others, were inversely correlated with fecal SCFA concentrations. Nearly all OTUs that were significantly associated with butyrate excretion were also associated with excretion of acetate or propionate. No microbial groups were significantly associated with fecal isobutyrate concentrations (Figure 3).

Finally, we examined the magnitude of the associations of fecal SCFA levels and gut microbiota diversity with obesity, central obesity and hypertension, using multivariable-adjusted log-binomial regression models with robust variance. In our cohort, 30% of participants were obese (*N* = 132), 41% had central obesity (*N* = 180), and 59% had hypertension (*N* = 259). We found that individuals in the highest vs. lowest tertile of butyrate excretion had 1.95 (95% confidence interval: 1.74, 2.16) times higher prevalence of obesity, 1.54 (1.37, 1.71) times higher prevalence of central obesity, and 1.31 (1.14, 1.47) times higher prevalence of hypertension (Table 3). Associations for other SCFAs had similar magnitude (Table S5) and persisted after further adjustment for serum LBP levels (Table 3). Participants with higher gut microbiota diversity had lower prevalence of obesity (Table 3).

The findings from our entire sample were confirmed in sensitivity analyses in which we used a subset of 217 participants with no missing values who did not report smoking or use of pharmacological treatments, including metformin (Figure S5, Table S6).

## 4. Discussion

In our community-based sample of Colombian adults, higher SCFA levels in the stool were associated with indicators of lower gut microbiota diversity, and higher gut permeability (measured by LPS binding protein), systemic inflammation (measured by hs-CRP), glycemia, dyslipidemia, obesity, central obesity, and hypertension. All associations remained after adjustment for possible confounders, including dietary fiber, total calories and physical activity, and the findings were not explained by medication use. As such, our results appear to favor the hypothesis that greater excretion of SCFAs in stool is a marker of poor gut health and cardiometabolic dysregulation.

Previous cross-sectional studies have reported higher fecal SCFA concentrations in overweight or obese individuals compared to lean individuals [13,15,32,33,34]. Moreover, Turnbaugh et al. demonstrated that genetically obese mice (i.e., homozygous for a mutation in the leptin gene (*ob*/*ob*) that produces a stereotyped, fully penetrant obesity phenotype) compared to their lean siblings (*ob*/+ and +/+) have a gut microbiome composition that promotes obesity through excess SCFA production and increased energy availability [14]. Our study extends these findings by showing that higher fecal SCFAs are also associated with central obesity, hypertension, subclinical measures of cardiometabolic disease (e.g., inflammation, glycemia and dyslipidemia), as well as a measure of gut permeability (LPS binding protein).

Our findings on fecal excretion of SCFAs may not be generalizable to SCFAs measured in circulation, which may better represent SCFA production and absorption. Vogt and Wolever [35] eloquently demonstrated that rates of acetate absorption and excretion are inversely correlated. Intestinal absorption of SCFAs is a function of transit time [35] and expression of SCFA transporters in the gut epithelium [2]. Inhibition of SCFA production and absorption may result in diarrhea [36], which is consistent with our own results (Table S3).

The studies that have measured SCFAs in circulation, as compared to stool, have come to somewhat different conclusions with respect to the association of SCFAs with obesity and cardiometabolic health. Boets et al. found that obese individuals have lower concentrations of propionate and butyrate in plasma compared to lean individuals [37]. Furthermore, in a cross-sectional study of 18 obese women, Layden et al. found that serum concentrations of acetate, but not propionate or butyrate, were inversely associated with fasting and 2-h insulin levels and visceral adipose tissue [38].

Human trials and experimental murine models have shown that increasing SCFAs through a high-fiber diet or direct supplementation may have beneficial health effects. In a randomized trial of 43 type 2 diabetic patients, Zhao et al. found that a high-fiber diet vs. an isocaloric control diet increased fecal butyrate concentrations, while also improving fasting glucose, insulin, and HbA1c [39]. In mice, butyrate supplementation was shown to prevent insulin resistance and obesity [40], via a PPARγ-dependent switch from lipogenesis to fat oxidation [41], and to reduce high-fat diet-induced intestinal barrier dysfunction and metabolic alterations, including hepatic steatosis [42].

To the best of our knowledge, we are the first to show that higher fecal SCFA excretion is associated with a measure of gut permeability. LBP is a biomarker of bacterial-derived LPS translocation from the gut to the periphery and higher serum LBP levels are thought to reflect gut permeability [43] and metabolic endotoxemia [44]. In our study, serum LBP was positively correlated with adiposity and cardiometabolic risk factors. However, when further adjusted our models for LBP, associations were only modestly attenuated (Table 3), indicating this measure of gut barrier function does not fully explain the association of higher fecal SCFA excretion with excess adiposity and cardiometabolic dysfunction.

Nevertheless, we provide evidence that gut microbiota dysbiosis, gut permeability, and the excretion of fecal SCFAs may be biologically related. Previous studies have found that disruption of the intestinal barrier eliminates epithelial hypoxia, increasing the amount of oxygen emanating from the colonic surface [5,45], and favoring the expansion of facultative anaerobic pathobionts [46,47]. In our study, individuals with high fecal SCFAs, obesity, and cardiometabolic disease risk factors showed signs of dysbiosis, including lower gut microbiota diversity and higher abundance of disease-associated microbial taxa: *Enterobacter hormaechei* [46], *Haemophilus parainfluenzae* [46,48], *Streptococcus* [48], and *SMB53* [49]. On the other hand, participants with lower fecal SCFA excretion had a diverse microbiome, enriched in beneficial microbes including *Akkermansia muciniphila*, a taxon involved in maintaining the integrity of the mucin layer [50]; *Christensenellaceae*, *Methanobrevibacter* and *Oscillospira*, taxa associated with lower BMI [51,52,53] and weight reduction [54]; and *Alistipes* and *Bacteroides*, bacteria that have been purported to play a role in ameliorating obesity [55]. Thus, it is possible that differences in microbial diversity and relative abundance of key taxa, including pathobionts, may lead to gut mucosa inflammation and therefore less efficient SCFA absorption [56].

Our study had several strengths, including the large sample size of community-dwelling adults; a rich data set that allowed us to control for many potential confounders and to restrict our sample to those who did not use medications, thus limiting the potential for reverse causality. Limitations to our study include the cross-sectional design, which precluded inference into causal relationships; SCFAs were only measured in feces, not in serum; the indirect measurement of gut permeability using serum LBP; and the possibility of residual confounding by diet, as it was assessed only once through 24-h diet recalls for the majority of participants. Moreover, because this is an observational study, we cannot exclude the possibility of unmeasured confounding by other factors.

## 5. Conclusions

We presented a comprehensive analysis demonstrating that fecal SCFA excretion, which cannot be equated to SCFA production or absorption, was positively associated with measures of gut microbiome dysbiosis, gut permeability, and cardiometabolic dysregulation, as well as higher prevalence of obesity, central obesity, and hypertension. Gut microbiota richness and the differential abundance of many microbial taxa were also associated with fecal SCFA excretion, as was the intake of dietary fiber, highlighting the potential for SCFA modification through diet, pre- and pro-biotic interventions. In light of the contradictory findings on SCFAs and health, controlled human feeding trials that assess both fecal and circulating SCFAs are needed to test the hypothesis that the association of higher fecal SCFA concentrations with obesity and cardiometabolic dysregulation is due to less efficient absorption and utilization of these metabolites.

## Figures and Tables

**Table 1:** Characteristics of the study population.

Variables	All Data	Fecal Butyrate				OTU Richness			
		Tertile 1	Tertile 2	Tertile 3	*p*-Value	Tertile 1	Tertile 2	Tertile 3	*p*-Value
*n*	441	147	147	147		150	147	144	
Age (years)	41 ± 1	40 ± 1	41 ± 1	41 ± 1	0.58	40 ± 1	40 ± 1	42 ± 1	0.05 *
Sex (%males: %females)	48:52	37:63	50:50	57:43	0.002 *	54:46	51:49	37:63	0.01 *
**Diet**									
Calorie intake (kcal/day)	1930 ± 21	1854 ± 31	1957 ± 39	1980 ± 38	0.05 *	1938 ± 35	1960 ± 39	1893 ± 35	0.50
Fiber intake (g/day)	17.7 ± 0.2	16.6 ± 0.4	18.3 ± 0.4	18.2 ± 0.4	0.002 *	17.5 ± 0.4	18.1 ± 0.4	17.5 ± 0.4	0.62
**Physical activity**									
MET/min/week	5104 ± 263	4160 ± 343	5458 ± 548	5695 ± 446	0.13	5538 ± 475	5224 ± 438	4530 ± 452	0.53
**Adiposity**									
BMI (kg/m^2^)	27.9 ± 0.2	26.8 ± 0.3	27.5 ± 0.4	29.6 ± 0.4	<0.0001 *	28.6 ± 0.4	28.1 ± 0.4	27.1 ± 0.4	0.03 *
Body fat (%)	37.2 ± 0.3	36.3 ± 0.4	37.1 ± 0.5	38.1 ± 0.5	0.03 *	37.3 ± 0.5	36.6 ± 0.4	37.6 ± 0.4	0.29
Waist circumference (cm)	92.8 ± 0.6	89.3 ± 0.9	91.7 ± 1.1	97.3 ± 1.1	<0.0001 *	94.6 ± 1.1	93.2 ± 1.1	90.3 ± 1.1	0.01 *
**Blood chemistry**									
HDL (mg/dL)	46 ± 1	47 ± 1	46 ± 1	45 ± 1	0.32	43 ± 1	47 ± 1	48 ± 1	0.007 *
LDL (mg/dL)	115 ± 1	116 ± 2	115 ± 3	114 ± 2	0.88	114 ± 2	115 ± 2	116 ± 2	0.84
VLDL (mg/dL)	28.7 ± 0.9	27.1 ± 1.7	28.3 ± 1.7	30.8 ± 1.5	0.05 *	31.2 ± 1.7	27.2 ± 1.4	27.7 ± 1.8	0.05 *
Triglycerides (mg/dL)	143 ± 5	135 ± 9	141 ± 8	154 ± 7	0.05 *	157 ± 8	136 ± 7	137 ± 9	0.03 *
hs-CRP (mg/L)	3.2 ± 0.2	2.44 ± 0.19	3.08 ± 0.36	3.93 ± 0.50	0.003 *	3.77 ± 0.50	3.15 ± 0.35	2.51 ± 0.21	0.05 *
Glucose (mg/dL)	89 ± 1	87 ± 2	90 ± 2	91 ± 2	0.07 *	89 ± 1	90 ± 2	89 ± 2	0.73
HbA1c (%)	5.55 ± 0.03	5.49 ± 0.05	5.58 ± 0.04	5.58 ± 0.05	0.25	5.49 ± 0.04	5.54 ± 0.06	5.63 ± 0.05	0.10
Insulin (µU/mL)	13.3 ± 0.4	12.0 ± 0.5	12.7 ± 0.7	15.1 ± 0.8	0.004 *	14.3 ± 0.7	12.8 ± 0.7	12.6 ± 0.7	0.07 *
HOMA-IR	3.12 ± 0.15	2.81 ± 0.16	3.31 ± 0.37	3.25 ± 0.18	0.06 *	3.01 ± 0.18	3.42 ± 0.36	2.85 ± 0.19	0.29
Leptin (ng/mL)	7.14 ± 0.30	7.01 ± 0.56	6.68 ± 0.51	7.72 ± 0.51	0.20	7.37 ± 0.54	7.16 ± 0.54	6.88 ± 0.48	0.84
Adiponectin (µg/mL)	6.8 ± 0.2	6.6 ± 0.3	7.3 ± 0.4	6.5 ± 0.3	0.08 *	6.3 ± 0.3	6.7 ± 0.3	7.4 ± 0.3	0.04 *
LBP (µg/mL)	4.50 ± 0.08	4.37 ± 0.13	4.56 ± 0.14	4.58 ± 0.13	0.48	4.84 ± 0.14	4.35 ± 0.13	4.31 ± 0.13	0.007 *
**Blood pressure**									
Systolic (mm Hg)	124 ± 1	120 ± 1	124 ± 2	130 ± 2	<0.0001 *	128 ± 2	124 ± 1	121 ± 1	0.002 *
Diastolic (mm Hg)	80 ± 1	78 ± 1	79 ± 1	84 ± 1	<0.0001 *	82 ± 1	79 ± 1	79 ± 1	0.03 *
Mean (mm Hg)	95 ± 1	92 ± 1	94 ± 1	99 ± 1	<0.0001 *	98 ± 1	94 ± 1	93 ± 1	0.01 *
**OTU richness**	144 ± 2	158 ± 3	143 ± 3	133 ± 3	<0.0001 *	103 ± 2	145 ± 1	186 ± 1	<0.0001 *
**Fecal SCFAs**									
Total SCFAs (µmol/g)	5.60 ± 0.36	1.22 ± 0.10	4.19 ± 0.25	11.39 ± 0.83	<0.0001 *	7.02 ± 0.57	5.06 ± 0.43	4.67 ± 0.79	<0.0001 *
Acetate (µmol/g)	3.83 ± 0.24	0.94 ± 0.08	3.00 ± 0.22	7.55 ± 0.53	<0.0001 *	4.56 ± 0.40	3.53 ± 0.31	3.38 ± 0.50	0.0004 *
Propionate (µmol/g)	1.18 ± 0.10	0.19 ± 0.02	0.81 ± 0.06	2.54 ± 0.24	<0.0001 *	1.66 ± 0.15	1.00 ± 0.10	0.88 ± 0.22	<0.0001 *
Butyrate (µmol/g)	0.59 ± 0.04	0.09 ± 0.01	0.38 ± 0.01	1.29 ± 0.11	<0.0001 *	0.80 ± 0.07	0.54 ± 0.04	0.41 ± 0.10	<0.0001 *
Isobutyrate (µmol/g)	0.04 ± 0.01	0.01 ± 0.001	0.03 ± 0.002	0.08 ± 0.02	<0.0001 *	0.03 ± 0.003	0.03 ± 0.003	0.05 ± 0.02	0.25

Variables presented overall and according to tertiles (tertile 1: Low; tertile 2: Intermediate; and tertile 3: High) of unadjusted fecal butyrate concentrations and gut microbiota diversity. Data are presented as mean ± SEM. *p*-values are from ANOVA, with the exception of sex (chi-squared test). FDR-adjusted *p*-values are highlighted (*: *q* < 0.05).

**Table 2:** Multivariable-adjusted Pearson correlations between measures of adiposity, cardiometabolic health, gut microbiota diversity, gut permeability, and fecal SCFA levels.

	Total SCFAs	Acetate	Propionate	Butyrate	Isobutyrate	OTU Richness
**Adiposity**						
BMI	0.28 *	0.26 *	0.29 *	0.25 *	0.13 *	−0.11 *
Body fat	0.13 *	0.12 *	0.14 *	0.11 *	0.09	−0.06
Waist circumference	0.26 *	0.23 *	0.28 *	0.24 *	0.14 *	−0.19 *
**Blood chemistry**						
HDL	−0.10 *	−0.08	−0.13 *	−0.11 *	−0.06	0.15 *
LDL	−0.02	−0.02	−0.01	0.00	0.01	0.03
VLDL	0.14 *	0.12 *	0.18 *	0.14 *	0.02	−0.15 *
Triglycerides	0.14 *	0.12 *	0.18 *	0.15 *	0.02	−0.16 *
hs-CRP	0.18 *	0.16 *	0.19 *	0.24 *	0.14 *	−0.11 *
Glucose	0.10	0.10	0.12 *	0.04	0.05	−0.06
HbA1c	0.12 *	0.14 *	0.10	0.06	0.11 *	0.02
Insulin	0.18 *	0.16 *	0.19 *	0.17 *	0.11 *	−0.10
HOMA-IR	0.09	0.07	0.09	0.08	0.04	−0.06
Leptin	0.06	0.06	0.07	0.06	0.06	0.01
Adiponectin	−0.05	−0.04	−0.07	−0.06	−0.01	0.13 *
LBP	0.18 *	0.17 *	0.17 *	0.17 *	0.07	−0.12 *
**Blood pressure**						
Systolic	0.22 *	0.19 *	0.25 *	0.23 *	0.07	−0.21 *
Diastolic	0.19 *	0.16 *	0.23 *	0.21 *	0.04	−0.16 *
Mean	0.21 *	0.18 *	0.25 *	0.23 *	0.06	−0.19 *
**OTU richness**	−0.28 *	−0.21 *	−0.35 *	−0.33 *	−0.06	—
**Fecal SCFAs**						
Total SCFAs	—	0.98 *	0.93 *	0.86 *	0.43 *	−0.28 *
Acetate	0.98 *	—	0.88 *	0.78 *	0.39 *	−0.21 *
Propionate	0.93 *	0.88 *	—	0.86 *	0.41 *	−0.35 *
Butyrate	0.86 *	0.78 *	0.86 *	—	0.51 *	−0.33 *
Isobutyrate	0.43 *	0.39 *	0.41 *	0.51 *	—	−0.06

Variables adjusted for age, city of origin, caloric intake, physical activity, and fiber intake. FDR-adjusted *p*-values are highlighted (* *q* < 0.05).

**Table 3:** Prevalence ratios of fecal butyrate concentrations and gut microbiota diversity for obesity, central obesity, and hypertension with robust 95% confidence intervals according to tertiles of fecal butyrate and OTU richness, a measures of gut microbiota diversity.

	Fecal Butyrate			OTU Richness		
	Tertile 1	Tertile 2	Tertile 3	Tertile 1	Tertile 2	Tertile 3
**Obesity ^1^**	*N* = 80	*N* = 93	*N* = 90	*N* = 80	*N* = 86	*N* = 97
Unadjusted model	Referent	1.12 (0.91, 1.33)	1.72 (1.51, 1.93)	Referent	0.91 (0.69, 1.12)	0.75 (0.55, 0.95)
Confounder-adjusted model ^4^	Referent	1.22 (1.02, 1.43)	1.95 (1.74, 2.16)	Referent	0.98 (0.77, 1.20)	0.72 (0.52, 0.93)
Confounder-adjusted ^4^ + LBP model	Referent	1.08 (0.88, 1.29)	1.73 (1.52, 1.94)	Referent	1.05 (0.84, 1.27)	0.78 (0.58, 0.98)
**Central obesity ^2^**	*N* = 144	*N* = 143	*N* = 144	*N* = 144	*N* = 144	*N* = 143
Unadjusted model	Referent	1.22 (1.06, 1.39)	1.56 (1.40, 1.73)	Referent	0.95 (0.78, 1.12)	0.96 (0.79, 1.12)
Confounder-adjusted model ^4^	Referent	1.22 (1.06, 1.39)	1.54 (1.37, 1.71)	Referent	0.97 (0.80, 1.13)	0.92 (0.75, 1.09)
Confounder-adjusted ^4^ + LBP model	Referent	1.10 (0.93, 1.27)	1.39 (1.22, 1.55)	Referent	0.99 (0.82, 1.15)	0.97 (0.80, 1.14)
**Hypertension ^3^**	*N* = 144	*N* = 143	*N* = 144	*N* = 144	*N* = 144	*N* = 143
Unadjusted model	Referent	1.16 (0.99, 1.32)	1.33 (1.16, 1.49)	Referent	0.91 (0.74, 1.07)	0.98 (0.82, 1.15)
Confounder-adjusted model ^4^	Referent	1.12 (0.96, 1.29)	1.31 (1.14, 1.47)	Referent	0.92 (0.76, 1.09)	0.89 (0.73, 1.06)
Confounder-adjusted ^4^ + LBP model	Referent	1.08 (0.91, 1.24)	1.25 (1.08, 1.42)	Referent	0.92 (0.76, 1.09)	0.90 (0.73, 1.06)

^1^ Defined as BMI ≥ 30 kg/m^2^; ^2^ defined as waist circumference ≥102 cm (men) and ≥88 cm (women); ^3^ defined as SBP ≥ 130 mm Hg or DBP ≥ 80 mm Hg or previous diagnosis of hypertension or use of antihypertensive medications; ^4^ Model adjusted for participant age, city of residence, physical activity, fiber intake, and total caloric intake.

## Supplementary Materials

The following are available online at http://www.mdpi.com/2072-6643/11/1/51/s1. Figure S1: Distribution of the gut microbiota diversity according to multivariable-adjusted fecal SCFA concentrations; Figure S2: Distribution of cardiometabolic health indicators according to multivariable-adjusted fecal butyrate concentration; Figure S3: Distribution of cardiometabolic health indicators according to multivariable-adjusted gut microbiota diversity; Figure S4: Principal coordinates analysis (PCoA) plot based on weighted UniFrac distances; Figure S5: Heatmap showing the correlations between rarefied OTU abundances and multivariable-adjusted fecal SCFA concentrations in the subset of participants who did not report smoking or use of pharmacological treatments; Table S1: Parameters of the single-ion monitoring (SIM) mode acquisition and performance of the SCFA quantification method using GC/MS; Table S2: Characteristics of the study population overall and according to tertiles of unadjusted fecal SCFA levels; Table S3: Multivariable-adjusted fecal SCFA concentrations according to stool consistency; Table S4: Procrustes analysis correlating the gut microbiota-beta diversity with SCFA concentrations and variables informing about the risk of cardiometabolic disease; Table S5: Prevalence ratios of total fecal SCFAs, acetate and propionate concentrations for obesity, central obesity, and hypertension with robust 95% confidence intervals; and Table S6: Prevalence ratios of fecal butyrate and total SCFA concentrations for obesity, central obesity, and hypertension with robust 95% confidence intervals in the subset of participants who did not report smoking or use of pharmacological treatments.

## References

[1] Flint H.J., Scott K.P., Duncan S.H., Louis P., Forano E. (2012). Microbial degradation of complex carbohydrates in the gut. Gut Microbes.

[2] Stumpff F. (2018). A look at the smelly side of physiology: Transport of short chain fatty acids. Pflugers Arch. Eur. J. Physiol..

[3] Hamer H.M., Jonkers D., Venema K., Vanhoutvin S., Troost F.J., Brummer R.J. (2008). Review article: The role of butyrate on colonic function. Aliment. Pharmacol. Ther..

[4] Den Besten G., Lange K., Havinga R., van Dijk T.H., Gerding A., van Eunen K., Muller M., Groen A.K., Hooiveld G.J., Bakker B.M. (2013). Gut-derived short-chain fatty acids are vividly assimilated into host carbohydrates and lipids. AJP Gastrointest. Liver Physiol..

[5] Kelly C.J., Zheng L., Campbell E.L., Saeedi B., Scholz C.C., Bayless A.J., Wilson K.E., Glover L.E., Kominsky D.J., Magnuson A. (2015). Crosstalk between microbiota-derived short-chain fatty acids and intestinal epithelial HIF augments tissue barrier function. Cell Host Microbe.

[6] Peng L., Li Z.-R., Green R.S., Holzman I.R., Lin J. (2009). Butyrate enhances the intestinal barrier by facilitating tight junction assembly via activation of AMP-activated protein kinase in Caco-2 cell monolayers. J. Nutr..

[7] Jung T.H., Park J.H., Jeon W.M., Han K.S. (2015). Butyrate modulates bacterial adherence on LS174T human colorectal cells by stimulating mucin secretion and MAPK signaling pathway. Nutr. Res. Pract..

[8] Cani P.D., Bibiloni R., Knauf C., Neyrinck A.M., Delzenne N.M. (2008). Changes in gut microbiota control metabolic diet-induced obesity and diabetes in mice. Diabetes.

[9] Byrne C.S., Chambers E.S., Morrison D.J., Frost G. (2015). The role of short chain fatty acids in appetite regulation and energy homeostasis. Int. J. Obes..

[10] Vieira A.T., MacIa L., Galvão I., Martins F.S., Canesso M.C.C., Amaral F.A., Garcia C.C., Maslowski K.M., De Leon E., Shim D. (2015). A role for gut microbiota and the metabolite-sensing receptor GPR43 in a murine model of gout. Arthritis Rheumatol..

[11] Smith P.M., Howitt M.R., Panikov N., Michaud M., Gallini C.A., Bohlooly Y.M., Glickman J.N., Garrett W.S., Bohlooly Y.M., Glickman J.N. (2013). The microbial metabolites, short-chain fatty acids, regulate colonic Treg cell homeostasis. Science.

[12] McNeil N.I. (1984). The contribution of the large intestine to energy supplies in man. Am. J. Clin. Nutr..

[13] Rahat-Rozenbloom S., Fernandes J., Gloor G.B., Wolever T.M.S. (2014). Evidence for greater production of colonic short-chain fatty acids in overweight than lean humans. Int. J. Obes..

[14] Turnbaugh P.J., Ley R.E., Mahowald M.A., Magrini V., Mardis E.R., Gordon J.I. (2006). An obesity-associated gut microbiome with increased capacity for energy harvest. Nature.

[15] Schwiertz A., Taras D., Schafer K., Beijer S., Bos N.A., Donus C., Hardt P.D., Schäfer K., Beijer S., Bos N.A. (2010). Microbiota and SCFA in lean and overweight healthy subjects. Obesity.

[16] Jumpertz R., Le D.S., Turnbaugh P.J., Trinidad C., Bogardus C., Gordon J.I., Krakoff J. (2011). Energy-balance studies reveal associations between gut microbes, caloric load, and nutrient absorption in humans. Am. J. Clin. Nutr..

[17] Turnbaugh P.J., Hamady M., Yatsunenko T., Cantarel B.L., Duncan A., Ley R.E., Sogin M.L., Jones W.J., Roe B.A., Affourtit J.P. (2009). A core gut microbiome in obese and lean twins. Nature.

[18] Zweigner J., Schumann R.R., Weber J.R. (2006). The role of lipopolysaccharide-binding protein in modulating the innate immune response. Microbes Infect..

[19] Siri W. (1961). Body Composition from Fluid Spaces and Density: Analysis of Methods.

[20] IPAQ Research Committee Guidelines for Data Processing and Analysis of the International Physical Activity Questionnaire (IPAQ): Short and Long Forms. http://www.academia.edu/5346814/Guidelines_for_Data_Processing_and_Analysis_of_the_International_Physical_Activity_Questionnaire_IPAQ_Short_and_Long_Forms_Contents.

[21] De la Cuesta-Zuluaga J., Mueller N.T., Corrales-Agudelo V., Velásquez-Mejía E.P., Carmona J.A., Abad J.M., Escobar J.S. (2017). Metformin is associated with higher relative abundance of mucin-degrading Akkermansia muciniphila and several short-chain fatty acid–producing microbiota in the gut. Diabetes Care.

[22] Schloss P.D., Westcott S.L., Ryabin T., Hall J.R., Hartmann M., Hollister E.B., Ryan A., Oakley B.B., Parks D.H., Courtney J. (2009). Introducing mothur: Open-source, platform-independent, community-supported software for describing and comparing microbial communities. Appl. Environ. Microbiol..

[23] DeSantis T.Z., Hugenholtz P., Larsen N., Rojas M., Brodie E.L., Keller K., Huber T., Dalevi D., Hu P., Andersen G.L. (2006). Greengenes, a chimera-checked 16S rRNA gene database and workbench compatible with ARB. Appl. Environ. Microbiol..

[24] Kindt R., Coe R. (2005). Tree Diversity Analysis. A Manual and Software for Common Statistical Methods for Ecological and Biodiversity Studies.

[25] Chen J., Bittinger K., Charlson E.S., Hoffmann C., Lewis J., Wu G.D., Collman R.G., Bushman F.D., Li H. (2012). Associating microbiome composition with environmental covariates using generalized UniFrac distances. Bioinformatics.

[26] Storey J.D., Bass A.J., Dabney A., Robinson D. qvalue: Q-Value Estimation for False Discovery Rate Control. http://github.com/jdstorey/qvalue.

[27] Claesson M.J., Cusack S., O’Sullivan O., Greene-Diniz R., de Weerd H., Flannery E., Marchesi J.R., Falush D., Dinan T., Fitzgerald G. (2011). Composition, variability, and temporal stability of the intestinal microbiota of the elderly. Proc. Natl. Acad. Sci. USA.

[28] De la Cuesta-Zuluaga J., Corrales-Agudelo V., Velásquez-Mejía E.P., Carmona J.A., Abad J.M., Escobar J.S. (2018). Gut microbiota is associated with obesity and cardiometabolic disease in a population in the midst of Westernization. Sci. Rep..

[29] Clarke S.F., Murphy E.F., O’Sullivan O., Lucey A.J., Humphreys M., Hogan A., Hayes P., O’Reilly M., Jeffery I.B., Wood-Martin R. (2014). Exercise and associated dietary extremes impact on gut microbial diversity. Gut.

[30] Peres-Neto P.R., Jackson D.A. (2001). How well do multivariate data sets match? The advantages of a procrustean superimposition approach over the Mantel test. Oecologia.

[31] Forslund K., Hildebrand F., Nielsen T., Falony G., Le Chatelier E., Sunagawa S., Prifti E., Vieira-Silva S., Gudmundsdottir V., Krogh Pedersen H. (2015). Disentangling type 2 diabetes and metformin treatment signatures in the human gut microbiota. Nature.

[32] Fernandes J., Su W., Rahat-Rozenbloom S., Wolever T.M., Comelli E.M. (2014). Adiposity, gut microbiota and faecal short chain fatty acids are linked in adult humans. Nutr Diabetes.

[33] Teixeira T.F.S., Grześkowiak Ł., Franceschini S.C.C., Bressan J., Ferreira C.L.L.F., Peluzio M.C.G. (2013). Higher level of faecal SCFA in women correlates with metabolic syndrome risk factors. Br. J. Nutr..

[34] Ppatil D., Pdhotre D., Gchavan S., Sultan A., Jain D.S., Lanjekar V.B., Gangawani J., Sshah P., Stodkar J., Shah S. (2012). Molecular analysis of gut microbiota in obesity among Indian individuals. J. Biosci..

[35] Vogt J.A., Wolever T.M. (2003). Fecal acetate is inversely related to acetate absorption from the human rectum and distal colon. J. Nutr..

[36] Binder H.J. (2010). Role of colonic short-chain fatty acid transport in diarrhea. Annu. Rev. Physiol..

[37] Boets E., Deroover L., Houben E., Vermeulen K., Gomand S.V., Delcour J.A., Verbeke K. (2015). Quantification of in vivo colonic short chain fatty acid production from inulin. Nutrients.

[38] Layden B.T., Yalamanchi S.K., Wolever T.M., Dunaif A., Lowe W.L. (2012). Negative association of acetate with visceral adipose tissue and insulin levels. Diabetes Metab. Syndr. Obes..

[39] Zhao L., Zhang F., Ding X., Wu G., Lam Y.Y., Shi Y., Shen Q., Dong W., Liu R., Ling Y. (2018). Gut bacteria selectively promoted by dietary fibers alleviate type 2 diabetes. Science.

[40] Gao Z., Yin J., Zhang J., Ward R.E., Martin R.J., Lefevre M., Cefalu W.T., Ye J. (2009). Butyrate improves insulin sensitivity and increases energy expenditure in mice. Diabetes.

[41] Den Besten G., Bleeker A., Gerding A., van Eunen K., Havinga R., van Dijk T.H., Oosterveer M.H., Jonker J.W., Groen A.K., Reijngoud D.-J. (2015). Short-chain fatty acids protect against high-fat diet-induced obesity via a PPARγ-dependent switch from lipogenesis to fat oxidation. Diabetes.

[42] Matheus V.A., Monteiro L.C.S., Oliveira R.B., Maschio D.A., Collares-Buzato C.B. (2017). Butyrate reduces high-fat diet-induced metabolic alterations, hepatic steatosis and pancreatic beta cell and intestinal barrier dysfunctions in prediabetic mice. Exp. Biol. Med..

[43] Guo S., Al-Sadi R., Said H.M., Ma T.Y. (2013). Lipopolysaccharide causes an increase in intestinal tight junction permeability in vitro and in vivo by inducing enterocyte membrane expression and localization of TLR-4 and CD14. Am. J. Pathol..

[44] Cani P.D., Amar J., Iglesias M.A., Poggi M., Knauf C., Bastelica D., Neyrinck A.M., Fava F., Tuohy K.M., Chabo C. (2007). Metabolic endotoxemia initiates obesity and insulin resistance. Diabetes.

[45] Albenberg L., Esipova T.V., Judge C.P., Bittinger K., Chen J., Laughlin A., Grunberg S., Baldassano R.N., Lewis J.D., Li H. (2014). Correlation between intraluminal oxygen gradient and radial partitioning of intestinal microbiota. Gastroenterology.

[46] Shin N.R., Whon T.W., Bae J.W. (2015). Proteobacteria: Microbial signature of dysbiosis in gut microbiota. Trends Biotechnol..

[47] Litvak Y., Byndloss M.X., Tsolis R.M., Bäumler A.J. (2017). Dysbiotic Proteobacteria expansion: A microbial signature of epithelial dysfunction. Curr. Opin. Microbiol..

[48] Huttenhower C., Gevers D., Knight R., Abubucker S., Badger J.H., Chinwalla A.T., Creasy H.H., Earl A.M., FitzGerald M.G., The Human Microbiome Project Consortium (2012). Structure, function and diversity of the healthy human microbiome. Nature.

[49] Napolitano A., Miller S., Nicholls A.W., Baker D., Van Horn S., Thomas E., Rajpal D., Spivak A., Brown J.R., Nunez D.J. (2014). Novel gut-based pharmacology of metformin in patients with type 2 diabetes mellitus. PLoS ONE.

[50] Everard A., Belzer C., Geurts L., Ouwerkerk J.P., Druart C., Bindels L.B., Guiot Y., Derrien M., Muccioli G.G., Delzenne N.M. (2013). Cross-talk between Akkermansia muciniphila and intestinal epithelium controls diet-induced obesity. Proc. Natl. Acad. Sci. USA.

[51] Million M., Angelakis E., Maraninchi M., Henry M., Giorgi R., Valero R., Vialettes B., Raoult D. (2013). Correlation between body mass index and gut concentrations of *Lactobacillus reuteri*, *Bifidobacterium animalis*, *Methanobrevibacter smithii* and *Escherichia coli*. Int. J. Obes..

[52] Goodrich J.K., Waters J.L., Poole A.C., Sutter J.L., Koren O., Blekhman R., Beaumont M., Van Treuren W., Knight R., Bell J.T. (2014). Human genetics shape the gut microbiome. Cell.

[53] Konikoff T., Gophna U. (2016). Oscillospira: A central, enigmatic component of the human gut microbiota. Trends Microbiol..

[54] Louis S., Tappu R.M., Damms-Machado A., Huson D.H., Bischoff S.C. (2016). Characterization of the gut microbial community of obese patients following a weight-loss intervention using whole metagenome shotgun sequencing. PLoS ONE.

[55] Ridaura V.K., Faith J.J., Rey F.E., Cheng J., Duncan A.E., Kau L., Griffi N.W., Lombard V., Henrissat B., Bain J.R. (2013). Gut microbiota from twins discordant for obesity modulate metabolism in mice. Science.

[56] Bäumler A.J., Sperandio V. (2016). Interactions between the microbiota and pathogenic bacteria in the gut. Nature.

