# Supplementary material for: Higher Fecal Short-Chain Fatty Acid Levels Are Associated with Gut Microbiome Dysbiosis, Obesity, Hypertension and Cardiometabolic Disease Risk Factors
Source: Nutrients. 2018 Dec 27;11(1):51. doi: 10.3390/nu11010051 (PMC6356834; doi:10.3390/nu11010051)
Supplement: Supplementary file 1 [file nutrients-11-00051-s001.pdf]

**Table S1.** Parameters of the single-ion monitoring (SIM) mode acquisition and performance of the short-chain fatty acid (SCFA) quantification method using gas chromatography–mass spectrometry (GC/MS).

| Descriptor | SCFAs | | | |
|---|---|---|---|---|
| | **Acetic** | **Propionic** | **Butyric** | **Isobutyric** |
| **SIM Ions m/z (m/z)/Dwell time (mS)** | | | | |
| SIM target | 43.1/30 | 74.1/25 | 60.1/30 | 43.1/30 |
| SIM qualifier | 60.1/15; 45.2/15 | 73.1/10; 45.1/15 | 88.2/15; 73.2/10 | 88.2/10; 73.1/15 |
| **Regression equation n=9** | | | | |
| (Y=mX+b) range=25-750 ng/mL | y=6E+07x+4E+06 | y=7E+07x+1E+07 | y=3E+08x+5E+07 | y=2E+08x+5E+07 |
| **Regression adjustment (r)** | 0.992 | 0.996 | 0.998 | 0.993 |
| **Response factor n=9** | | | | |
| (RF+%RSD) | 1.09E-08 ≤ 2.0 | 7.40E-09 ≤ 2.0 | 1.75E-09 ≤ 2.0 | 1.90E-09 ≤ 2.0 |
| **Linearity test** | | | | |
| Homoscedasticity value ($G_{exp}$<$G_{table}$) | 0.4924 | 0.0947 | 0.0942 | 0.1478 |
| $G_{table}$ (a=0.05; k=6; n=3)=0.616) | | | | |
| **Precision (n=27)** | | | | |
| Instrument (Repeatability, %RSD <3) | 1.72 | 0.99 | 1.12 | 1.09 |
| Method (%RSD <2) | 1.32 | 1.04 | 1.11 | 1.08 |
| **Recovery (n=9)** | | | | |
| % ± SD | 99.45 ± 1.22 | 100.45 ± 1.04 | 99.92 ± 0.99 | 99.84 ± 1.02 |
| Cochran test value ($G_{exp}$<$G_{table}$) | 0.637 | 0.211 | 0.206 | 0.198 |
| $G_{table}$ (a=0.05; k=3; n=3)=0.871 | | | | |

**Table S2.** Characteristics of the study population overall and according to tertiles (1: low, 2: intermediate, 3: high) of unadjusted fecal SCFA levels (total SCFAs, acetate, propionate and isobutyrate). Data presented as mean ± SEM. P-values from ANOVA to the exception of sex (chi-squared test). FDR-adjusted p-values are highlighted (*=*q*<0.05).

| Variables | Total fecal SCFAs (µmol/g) | | | | Fecal acetate (µmol/g) | | | |
|---|---|---|---|---|---|---|---|---|
| | **Tertile 1** | **Tertile 2** | **Tertile 3** | **P-value** | **Tertile 1** | **Tertile 2** | **Tertile 3** | **P-value** |
| n | 147 | 147 | 147 | | 147 | 147 | 147 | |
| Age (years) | 40 ± 1 | 41 ± 1 | 42 ± 1 | 0.40 | 39 ± 1 | 41 ± 1 | 42 ± 1 | 0.06* |
| Sex (%males:%females) | 40/60 | 50/50 | 54/46 | 0.05* | 41/59 | 51/49 | 52/48 | 0.10 |
| **Diet** | | | | | | | | |
| Calorie intake (kcal/day) | 1873 ± 32 | 1941 ± 40 | 1977 ± 37 | 0.14 | 1887 ± 34 | 1928 ± 38 | 1976 ± 38 | 0.10 |
| Fiber intake (g/day) | 16.7 ± 0.4 | 18.4 ± 0.4 | 17.9 ± 0.4 | 0.009* | 16.9 ± 0.4 | 18.0 ± 0.4 | 18.2 ± 0.4 | 0.02* |
| **Physical activity** | | | | | | | | |
| MET/min/week | 4308 ± 376 | 5456 ± 534 | 5550 ± 440 | 0.25 | 4349 ± 376 | 5507 ± 543 | 5457 ± 430 | 0.19 |
| **Adiposity** | | | | | | | | |
| BMI (kg/m$^2$) | 26.7 ± 0.3 | 27.5 ± 0.4 | 29.6 ± 0.5 | <0.0001* | 26.8 ± 0.3 | 27.2 ± 0.4 | 29.7 ± 0.5 | <0.0001* |
| Body fat (%) | 36.3 ± 0.4 | 37.1 ± 0.5 | 38.1 ± 0.5 | 0.02* | 36.4 ± 0.4 | 36.8 ± 0.5 | 38.3 ± 0.5 | 0.006* |
| Waist circumference (cm) | 89.4 ± 0.9 | 92.0 ± 1.1 | 96.9 ± 1.2 | <0.0001* | 89.7 ± 0.9 | 91.7 ± 1.0 | 96.8 ± 1.2 | <0.0001* |
| **Blood chemistry** | | | | | | | | |
| HDL (mg/dL) | 46 ± 1 | 46 ± 1 | 46 ± 1 | 0.98 | 46 ± 1 | 46 ± 1 | 47 ± 1 | 0.69 |
| LDL (mg/dL) | 117 ± 2 | 115 ± 2 | 113 ± 2 | 0.53 | 116 ± 2 | 116 ± 2 | 113 ± 2 | 0.47 |
| VLDL (mg/dL) | 27.3 ± 1.8 | 28.3 ± 1.4 | 30.6 ± 1.7 | 0.10 | 27.4 ± 1.8 | 29.0 ± 1.4 | 29.8 ± 1.6 | 0.08* |
| Triglycerides (mg/dL) | 136 ± 9 | 142 ± 7 | 152 ± 8 | 0.11 | 137 ± 9 | 144 ± 7 | 149 ± 8 | 0.08* |
| hs-CRP (mg/L) | 2.64 ± 0.32 | 3.41 ± 0.44 | 3.4 ± 0.36 | 0.03* | 2.72 ± 0.32 | 3.22 ± 0.43 | 3.50 ± 0.36 | 0.02* |
| Glucose (mg/dL) | 87 ± 2 | 90 ± 2 | 92 ± 2 | 0.02* | 86 ± 2 | 90 ± 2 | 92 ± 2 | 0.005* |
| HbA1c (%) | 5.46 ± 0.04 | 5.57 ± 0.06 | 5.63 ± 0.06 | 0.05* | 5.44 ± 0.04 | 5.58 ± 0.05 | 5.64 ± 0.06 | 0.006* |
| Insulin (µU/mL) | 12.0 ± 0.6 | 12.8 ± 0.7 | 15.0 ± 0.8 | 0.01* | 11.7 ± 0.5 | 13.2 ± 0.7 | 14.9 ± 0.8 | 0.005* |
| HOMA-IR | 3.07 ± 0.34 | 3.20 ± 0.23 | 3.10 ± 0.17 | 0.43 | 3.17 ± 0.35 | 3.17 ± 0.21 | 3.03 ± 0.17 | 0.36 |
| Leptin (ng/mL) | 6.92 ± 0.56 | 6.51 ± 0.49 | 7.98 ± 0.52 | 0.23 | 7.05 ± 0.58 | 6.15 ± 0.44 | 8.22 ± 0.54 | 0.17 |
| Adiponectin (µg/mL) | 6.4 ± 0.2 | 7.2 ± 0.4 | 6.8 ± 0.4 | 0.44 | 6.5 ± 0.2 | 7.0 ± 0.4 | 6.9 ± 0.4 | 0.53 |
| LBP (µg/mL) | 4.27 ± 0.14 | 4.66 ± 0.14 | 4.58 ± 0.12 | 0.09 | 4.26 ± 0.14 | 4.59 ± 0.13 | 4.66 ± 0.13 | 0.03* |
| **Blood pressure** | | | | | | | | |
| Systolic (mm Hg) | 120 ± 1 | 125 ± 2 | 128 ± 2 | 0.0005* | 120 ± 1 | 125 ± 2 | 128 ± 2 | 0.001* |
| Diastolic (mm Hg) | 78 ± 1 | 80 ± 1 | 83 ± 1 | 0.001* | 78 ± 1 | 80 ± 1 | 82 ± 1 | 0.001* |
| Mean (mm Hg) | 92 ± 1 | 95 ± 1 | 98 ± 1 | 0.0006* | 92 ± 1 | 95 ± 1 | 97 ± 1 | 0.001* |
| **OTU richness** | 157 ± 3 | 140 ± 3 | 136 ± 3 | <0.0001* | 154 ± 3 | 140 ± 3 | 140 ± 3 | 0.002* |
| **Fecal SCFAs** | | | | | | | | |
| Total SCFAs (µmol/g) | 0.87 ± 0.05 | 3.70 ± 0.08 | 12.24 ± 0.81 | <0.0001* | 0.93 ± 0.06 | 3.81 ± 0.12 | 12.06 ± 0.82 | <0.0001* |
| Acetate (µmol/g) | 0.60 ± 0.04 | 2.51 ± 0.07 | 8.38 ± 0.52 | <0.0001* | 0.57 ± 0.04 | 2.47 ± 0.06 | 8.45 ± 0.51 | <0.0001* |
| Propionate (µmol/g) | 0.15 ± 0.01 | 0.74 ± 0.03 | 2.66 ± 0.24 | <0.0001* | 0.21 ± 0.02 | 0.85 ± 0.07 | 2.49 ± 0.24 | <0.0001* |
| Butyrate (µmol/g) | 0.12 ± 0.01 | 0.45 ± 0.02 | 1.19 ± 0.11 | <0.0001* | 0.16 ± 0.02 | 0.48 ± 0.03 | 1.12 ± 0.11 | <0.0001* |
| Isobutyrate (µmol/g) | 0.01 ± 0.001 | 0.03 ± 0.002 | 0.07 ± 0.02 | <0.0001* | 0.01 ± 0.001 | 0.03 ± 0.002 | 0.07 ± 0.02 | <0.0001* |

**Table S2 (cont.)**

| Variables | Fecal propionate (μmol/g) | | | | Fecal isobutyrate (μmol/g) | | | |
|---|---|---|---|---|---|---|---|---|
| | Tertile 1 | Tertile 2 | Tertile 3 | P-value | Tertile 1 | Tertile 2 | Tertile 3 | P-value |
| n | 147 | 147 | 147 | | 147 | 147 | 147 | |
| Age (years) | 40 ± 1 | 41 ± 1 | 41 ± 1 | 0.34 | 40 ± 1 | 41 ± 1 | 40 ± 1 | 0.90 |
| Sex (%males:%females) | 41/59 | 48/52 | 55/45 | 0.06* | 42/58 | 56/44 | 46/54 | 0.06 |
| **Diet** | | | | | | | | |
| Calorie intake (kcal/day) | 1886 ± 32 | 1931 ± 41 | 1974 ± 36 | 0.10* | 1897 ± 39 | 1977 ± 37 | 1918 ± 33 | 0.55 |
| Fiber intake (g/day) | 16.8 ± 0.4 | 18.5 ± 0.4 | 17.9 ± 0.4 | 0.04* | 16.9 ± 0.4 | 18.7 ± 0.4 | 17.4 ± 0.4 | 0.42 |
| **Physical activity** | | | | | | | | |
| MET/min/week | 4059 ± 340 | 5671 ± 547 | 5583 ± 449 | 0.08* | 5197 ± 468 | 4621 ± 412 | 5494 ± 485 | 0.62 |
| **Adiposity** | | | | | | | | |
| BMI (kg/m$^2$) | 26.7 ± 0.3 | 27.5 ± 0.4 | 29.5 ± 0.5 | <0.0001* | 27.2 ± 0.4 | 27.8 ± 0.4 | 28.7 ± 0.4 | 0.01 |
| Body fat (%) | 36.0 ± 0.4 | 37.6 ± 0.4 | 37.9 ± 0.5 | 0.006* | 36.7 ± 0.5 | 36.7 ± 0.5 | 38.2 ± 0.4 | 0.02 |
| Waist circumference (cm) | 89.3 ± 0.9 | 91.8 ± 1.1 | 97.1 ± 1.2 | <0.0001* | 90.6 ± 1.0 | 93.1 ± 1.1 | 94.5 ± 1.2 | 0.02 |
| **Blood chemistry** | | | | | | | | |
| HDL (mg/dL) | 46 ± 1 | 46 ± 1 | 46 ± 1 | 0.66 | 46 ± 1 | 45 ± 1 | 46 ± 1 | 0.78 |
| LDL (mg/dL) | 116 ± 2 | 114 ± 2 | 114 ± 2 | 0.59 | 115 ± 2 | 115 ± 2 | 115 ± 2 | 0.99 |
| VLDL (mg/dL) | 26.9 ± 1.7 | 28.5 ± 1.4 | 30.8 ± 1.7 | 0.02* | 28.8 ± 1.8 | 28.9 ± 1.4 | 28.5 ± 1.6 | 0.93 |
| Triglycerides (mg/dL) | 134 ± 9 | 142 ± 7 | 153 ± 8 | 0.02* | 143 ± 9 | 144 ± 7 | 143 ± 8 | 0.98 |
| hs-CRP (mg/L) | 2.62 ± 0.32 | 3.12 ± 0.35 | 3.71 ± 0.45 | 0.005* | 2.52 ± 0.21 | 3.05 ± 0.35 | 3.88 ± 0.50 | 0.004 |
| Glucose (mg/dL) | 88 ± 2 | 87 ± 1 | 93 ± 2 | 0.002* | 88 ± 2 | 89 ± 1 | 91 ± 2 | 0.17 |
| HbA1c (%) | 5.50 ± 0.05 | 5.54 ± 0.04 | 5.62 ± 0.06 | 0.09* | 5.49 ± 0.04 | 5.57 ± 0.05 | 5.60 ± 0.06 | 0.12 |
| Insulin (μU/mL) | 12.0 ± 0.6 | 12.8 ± 0.7 | 15.0 ± 0.8 | 0.003* | 12.1 ± 0.5 | 13.2 ± 0.8 | 14.5 ± 0.8 | 0.02 |
| HOMA-IR | 2.88 ± 0.17 | 3.06 ± 0.35 | 3.43 ± 0.22 | 0.03* | 3.14 ± 0.34 | 3.02 ± 0.22 | 3.22 ± 0.19 | 0.23 |
| Leptin (ng/mL) | 6.83 ± 0.56 | 6.88 ± 0.50 | 7.71 ± 0.51 | 0.26 | 6.68 ± 0.45 | 6.92 ± 0.60 | 7.81 ± 0.51 | 0.16 |
| Adiponectin (μg/mL) | 6.5 ± 0.2 | 7.5 ± 0.4 | 6.4 ± 0.3 | 0.22 | 6.7 ± 0.3 | 6.6 ± 0.3 | 7.1 ± 0.4 | 0.66 |
| LBP (μg/mL) | 4.32 ± 0.13 | 4.56 ± 0.14 | 4.62 ± 0.12 | 0.12 | 4.37 ± 0.13 | 4.63 ± 0.13 | 4.51 ± 0.13 | 0.45 |
| **Blood pressure** | | | | | | | | |
| Systolic (mm Hg) | 121 ± 1 | 123 ± 2 | 129 ± 2 | <0.0001* | 123 ± 2 | 126 ± 1 | 124 ± 2 | 0.41 |
| Diastolic (mm Hg) | 78 ± 1 | 79 ± 1 | 83 ± 1 | 0.0004* | 80 ± 1 | 81 ± 1 | 80 ± 1 | 0.94 |
| Mean (mm Hg) | 92 ± 1 | 94 ± 1 | 99 ± 1 | 0.0001* | 94 ± 1 | 96 ± 1 | 95 ± 1 | 0.68 |
| **OTU richness** | 158 ± 3 | 144 ± 3 | 131 ± 3 | <0.0001* | 147 ± 3 | 141 ± 3 | 144 ± 3 | 0.51 |
| **Fecal SCFAs** | | | | | | | | |
| Total SCFAs (μmol/g) | 0.96 ± 0.07 | 3.90 ± 0.13 | 11.94 ± 0.82 | <0.0001* | 3.91 ± 0.51 | 3.89 ± 0.31 | 9.00 ± 0.82 | <0.0001 |
| Acetate (μmol/g) | 0.71 ± 0.06 | 2.77 ± 0.11 | 8.02 ± 0.54 | <0.0001* | 2.72 ± 0.35 | 2.60 ± 0.21 | 6.18 ± 0.52 | <0.0001 |
| Propionate (μmol/g) | 0.14 ± 0.01 | 0.69 ± 0.02 | 2.73 ± 0.24 | <0.0001* | 0.86 ± 0.13 | 0.82 ± 0.08 | 1.87 ± 0.23 | <0.0001 |
| Butyrate (μmol/g) | 0.11 ± 0.01 | 0.45 ± 0.03 | 1.20 ± 0.11 | <0.0001* | 0.33 ± 0.04 | 0.47 ± 0.04 | 0.95 ± 0.11 | <0.0001 |
| Isobutyrate (μmol/g) | 0.01 ± 0.001 | 0.03 ± 0.002 | 0.08 ± 0.02 | <0.0001* | 0.0002 ± 0.0001 | 0.02 ± 0.001 | 0.09 ± 0.02 | <0.0001 |

**Table S3.** Multivariable-adjusted fecal SCFA concentrations according to stool consistency. Models adjusted for participant age, city of residence, physical activity, fiber intake and caloric intake. Stool consistency sorted from rapid to slow intestinal transit time. P-values from ANOVA.

| Fecal SCFAs | Rapid transit time → Slow transit time | | | | |
|---|---|---|---|---|---|
| | Diarrheic (N=18) | Mushy (N=63) | Normal (N=293) | Hard (N=67) | P-value |
| Acetate | 0.48 ± 0.26 | 0.09 ± 0.14 | 0.02 ± 0.07 | -0.32 ± 0.18 | 0.06 |
| Propionate | 0.82 ± 0.29 | 0.06 ± 0.17 | 0.05 ± 0.07 | -0.48 ± 0.15 | 0.0003 |
| Butyrate | 0.78 ± 0.27 | 0.03 ± 0.18 | 0.09 ± 0.08 | -0.64 ± 0.18 | <0.0001 |
| Total SCFAs | 0.64 ± 0.29 | 0.07 ± 0.15 | 0.04 ± 0.07 | -0.43 ± 0.18 | 0.005 |
| Isobutyrate | -0.16 ± 0.43 | 0.24 ± 0.21 | 0.01 ± 0.10 | -0.22 ± 0.23 | 0.50 |

**Table S4.** Procrustes analysis correlating the gut microbiota-beta diversity with SCFA concentrations and variables informing about the risk of cardiometabolic disease. P-values obtained from 10,000 permutations. cor = correlation in a symmetric Procrustes rotation; the Y axis was scaled in all cases.

| Variable | Unweighted UniFrac | | | Weighted UniFrac | | |
|---|---|---|---|---|---|---|
| | cor | P-value | q-value | cor | P-value | q-value |
| Total SCFAs | 0.14 | 0.0001 | 0.0002 | 0.17 | 0.0001 | 0.0002 |
| Acetate | 0.12 | 0.0002 | 0.0003 | 0.15 | 0.0001 | 0.0002 |
| Propionate | 0.16 | 0.0001 | 0.0002 | 0.19 | 0.0001 | 0.0002 |
| Butyrate | 0.17 | 0.0001 | 0.0002 | 0.19 | 0.0001 | 0.0002 |
| Isobutyrate | 0.10 | 0.003 | 0.004 | 0.06 | 0.19 | 0.19 |
| Body mass index | 0.07 | 0.07 | 0.08 | 0.10 | 0.0007 | 0.001 |
| Waist circumference | 0.08 | 0.02 | 0.02 | 0.11 | 0.0002 | 0.0003 |
| Blood pressure | 0.09 | 0.006 | 0.008 | 0.12 | 0.0002 | 0.0003 |

**Table S5.** Prevalence ratios of total fecal SCFAs, acetate and propionate concentrations for obesity, central obesity, and hypertension with robust 95% confidence intervals. Levels of SCFAs were divided by tertiles (tertile 1: low level, tertile 2: intermediate level, tertile 3: high level).

| | **Total fecal SCFAs** | | |
|---|---|---|---|
| | **Tertile 1** | **Tertile 2** | **Tertile 3** |
| **Obesity**[1] | N=78 | N=81 | N=104 |
| Unadjusted model | Referent | 1.26 (1.04, 1.48) | 1.93 (1.74, 2.13) |
| Confounder-adjusted model[4] | Referent | 1.56 (1.33, 1.78) | 2.24 (2.04, 2.44) |
| Confounder-adjusted[4] + LBP model | Referent | 1.33 (1.10, 1.55) | 1.87 (1.67, 2.07) |
| **Central obesity**[2] | N=144 | N=143 | N=144 |
| Unadjusted model | Referent | 1.27 (1.10, 1.44) | 1.88 (1.72, 2.05) |
| Confounder-adjusted model[4] | Referent | 1.15 (0.98, 1.32) | 1.80 (1.63, 1.96) |
| Confounder-adjusted[4] + LBP model | Referent | 0.99 (0.82, 1.16) | 1.54 (1.37, 1.71) |
| **Hypertension**[3] | N=144 | N=143 | N=144 |
| Unadjusted model | Referent | 1.16 (0.99, 1.32) | 1.33 (1.16, 1.49) |
| Confounder-adjusted model[4] | Referent | 1.15 (0.99, 1.32) | 1.26 (1.10, 1.43) |
| Confounder-adjusted[4] + LBP model | Referent | 1.09 (0.93, 1.26) | 1.19 (1.03, 1.36) |

[1] Defined as BMI ≥30 kg/m$^2$; [2] defined waist circumference ≥102 cm (men) and ≥88 cm (women); [3] defined as SBP ≥130 mm Hg or DBP ≥80 mm Hg or previous diagnosis of hypertension or use of antihypertensive medications; [4] model adjusted for participant age, city of residence, physical activity, fiber intake and total caloric intake.

**Table S5 (cont.)**

| | **Fecal acetate** | | |
|---|---|---|---|
| | **Tertile 1** | **Tertile 2** | **Tertile 3** |
| **Obesity**[1] | N=77 | N=77 | N=109 |
| Unadjusted model | Referent | 1.15 (0.92, 1.38) | 1.80 (1.61, 2.00) |
| Confounder-adjusted model[4] | Referent | 1.43 (1.20, 1.66) | 2.04 (1.85, 2.24) |
| Confounder-adjusted[4] + LBP model | Referent | 1.30 (1.07, 1.53) | 1.76 (1.57, 1.96) |
| **Central obesity**[2] | N=144 | N=143 | N=144 |
| Unadjusted model | Referent | 1.12 (0.96, 1.29) | 1.84 (1.68, 2.01) |
| Confounder-adjusted model[4] | Referent | 0.98 (0.81, 1.15) | 1.72 (1.56, 1.89) |
| Confounder-adjusted[4] + LBP model | Referent | 0.86 (0.70, 1.03) | 1.48 (1.31, 1.64) |
| **Hypertension**[3] | N=144 | N=143 | N=144 |
| Unadjusted model | Referent | 1.06 (0.90, 1.23) | 1.33 (1.17, 1.50) |
| Confounder-adjusted model[4] | Referent | 1.06 (0.89, 1.22) | 1.27 (1.10, 1.43) |
| Confounder-adjusted[4] + LBP model | Referent | 1.02 (0.86, 1.19) | 1.20 (1.04, 1.36) |

[1] Defined as BMI ≥30 kg/m$^2$; [2] defined waist circumference ≥102 cm (men) and ≥88 cm (women); [3] defined as SBP ≥130 mm Hg or DBP ≥80 mm Hg or previous diagnosis of hypertension or use of antihypertensive medications; [4] model adjusted for participant age, city of residence, physical activity, fiber intake and total caloric intake.

**Table S5 (cont.)**

| | Fecal propionate | | |
|---|---|---|---|
| | **Tertile 1** | **Tertile 2** | **Tertile 3** |
| **Obesity**[1] | N=83 | N=84 | N=96 |
| Unadjusted model | Referent | 1.23 (1.01, 1.45) | 1.85 (1.64, 2.05) |
| Confounder-adjusted model[4] | Referent | 1.38 (1.16, 1.60) | 2.22 (2.01, 2.42) |
| Confounder-adjusted[4] + LBP model | Referent | 1.25 (1.03, 1.47) | 1.96 (1.76, 2.17) |
| **Central obesity**[2] | N=144 | N=143 | N=144 |
| Unadjusted model | Referent | 1.29 (1.12, 1.46) | 1.77 (1.60, 1.93) |
| Confounder-adjusted model[4] | Referent | 1.20 (1.04, 1.37) | 1.77 (1.60, 1.94) |
| Confounder-adjusted[4] + LBP model | Referent | 1.06 (0.90, 1.23) | 1.55 (1.38, 1.72) |
| **Hypertension**[3] | N=144 | N=143 | N=144 |
| Unadjusted model | Referent | 1.05 (0.88, 1.21) | 1.35 (1.18, 1.51) |
| Confounder-adjusted model[4] | Referent | 1.01 (0.85, 1.18) | 1.32 (1.16, 1.49) |
| Confounder-adjusted[4] + LBP model | Referent | 0.97 (0.81, 1.14) | 1.28 (1.11, 1.44) |

[1] Defined as BMI ≥30 kg/m$^2$; [2] defined waist circumference ≥102 cm (men) and ≥88 cm (women); [3] defined as SBP ≥130 mm Hg or DBP ≥80 mm Hg or previous diagnosis of hypertension or use of antihypertensive medications; [4] model adjusted for participant age, city of residence, physical activity, fiber intake and total caloric intake.

**Table S6.** Prevalence ratios of fecal butyrate and total SCFA concentrations for obesity, central obesity, and hypertension with robust 95% confidence intervals in the subset of participants who did not report smoking or use of pharmacological treatments. Levels of SCFAs were divided by tertiles (tertile 1: low level, tertile 2: intermediate level, tertile 3: high level).

| | **Fecal butyrate** | | |
|---|---|---|---|
| | **Tertile 1** | **Tertile 2** | **Tertile 3** |
| **Obesity**[1] | N=40 | N=45 | N=46 |
| Unadjusted model | Referent | 1.07 (0.75, 1.38) | 2.26 (1.96, 2.57) |
| Confounder-adjusted model[4] | Referent | 1.00 (0.68, 1.32) | 2.69 (2.38, 3.00) |
| Confounder-adjusted[4] + LBP model | Referent | 0.85 (0.53, 1.18) | 2.32 (2.00, 2.63) |
| **Central obesity**[2] | N=73 | N=72 | N=72 |
| Unadjusted model | Referent | 1.13 (0.89, 1.38) | 1.91 (1.67, 2.15) |
| Confounder-adjusted model[4] | Referent | 0.89 (0.64, 1.14) | 2.00 (1.76, 2.24) |
| Confounder-adjusted[4] + LBP model | Referent | 0.78 (0.53, 1.02) | 1.74 (1.50, 1.98) |
| **Hypertension**[3] | N=73 | N=72 | N=72 |
| Unadjusted model | Referent | 1.13 (0.89, 1.38) | 1.91 (1.67, 2.15) |
| Confounder-adjusted model[4] | Referent | 0.89 (0.64, 1.14) | 2.00 (1.76, 2.24) |
| Confounder-adjusted[4] + LBP model | Referent | 0.78 (0.53, 1.02) | 1.74 (1.50, 1.98) |

[1] Defined as BMI ≥30 kg/m$^2$; [2] defined waist circumference ≥102 cm (men) and ≥88 cm (women); [3] defined as SBP ≥130 mm Hg or DBP ≥80 mm Hg or previous diagnosis of hypertension or use of antihypertensive medications; [4] model adjusted for participant age, city of residence, physical activity, fiber intake and total caloric intake.

**Table S6 (cont.)**

| | **Total fecal SCFAs** | | |
|---|---|---|---|
| | **Tertile 1** | **Tertile 2** | **Tertile 3** |
| **Obesity**[1] | N=40 | N=38 | N=53 |
| Unadjusted model | Referent | 0.86 (0.51, 1.21) | 1.92 (1.64, 2.20) |
| Confounder-adjusted model[4] | Referent | 0.69 (0.32, 1.06) | 2.17 (1.88, 2.45) |
| Confounder-adjusted[4] + LBP model | Referent | 0.66 (0.30, 1.03) | 2.05 (1.77, 2.34) |
| **Central obesity**[2] | N=73 | N=72 | N=72 |
| Unadjusted model | Referent | 0.96 (0.72, 1.20) | 1.65 (1.42, 1.89) |
| Confounder-adjusted model[4] | Referent | 0.74 (0.50, 0.99) | 1.62 (1.38, 1.86) |
| Confounder-adjusted[4] + LBP model | Referent | 0.63 (0.38, 0.88) | 1.37 (1.13, 1.62) |
| **Hypertension**[3] | N=73 | N=72 | N=72 |
| Unadjusted model | Referent | 0.96 (0.72, 1.20) | 1.65 (1.42, 1.89) |
| Confounder-adjusted model[4] | Referent | 0.74 (0.50, 0.99) | 1.62 (1.38, 1.86) |
| Confounder-adjusted[4] + LBP model | Referent | 0.63 (0.38, 0.88) | 1.37 (1.13, 1.62) |

[1] Defined as BMI ≥30 kg/m$^2$; [2] defined waist circumference ≥102 cm (men) and ≥88 cm (women); [3] defined as SBP ≥130 mm Hg or DBP ≥80 mm Hg or previous diagnosis of hypertension or use of antihypertensive medications; [4] model adjusted for participant age, city of residence, physical activity, fiber intake and total caloric intake.

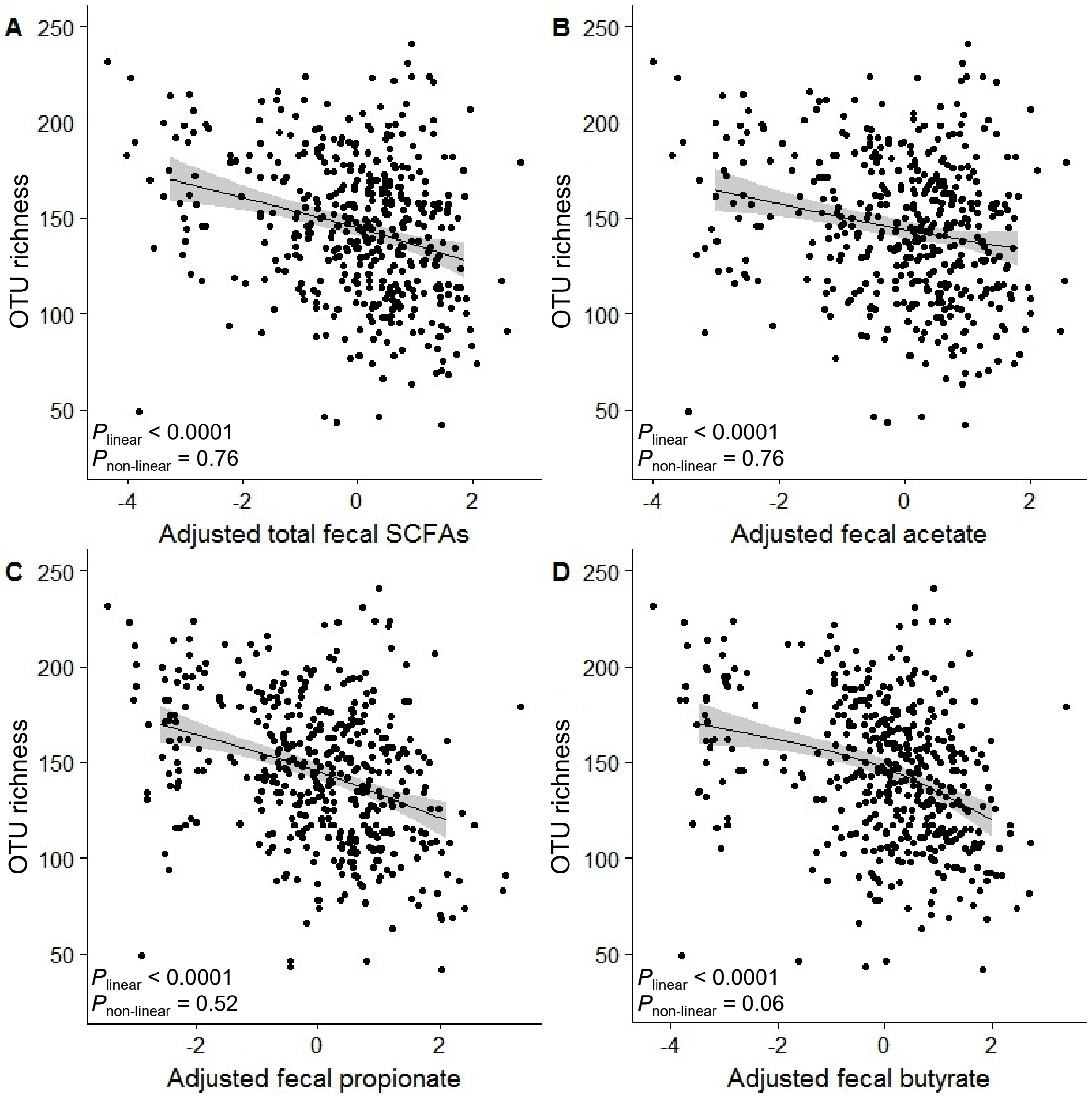


**Figure S1.** Distribution of the gut microbiota diversity according to multivariable-adjusted fecal SCFA concentrations (**A**: total SCFAs; **B**: acetate; **C**: propionate; **D**: butyrate). SCFA concentrations adjusted for age, city of origin, caloric intake, physical activity and fiber intake. Restricted cubic splines fits with 95% confidence intervals are shown. P-values from ordinary least squares testing the linearity and non-linearity of the regression are also shown.

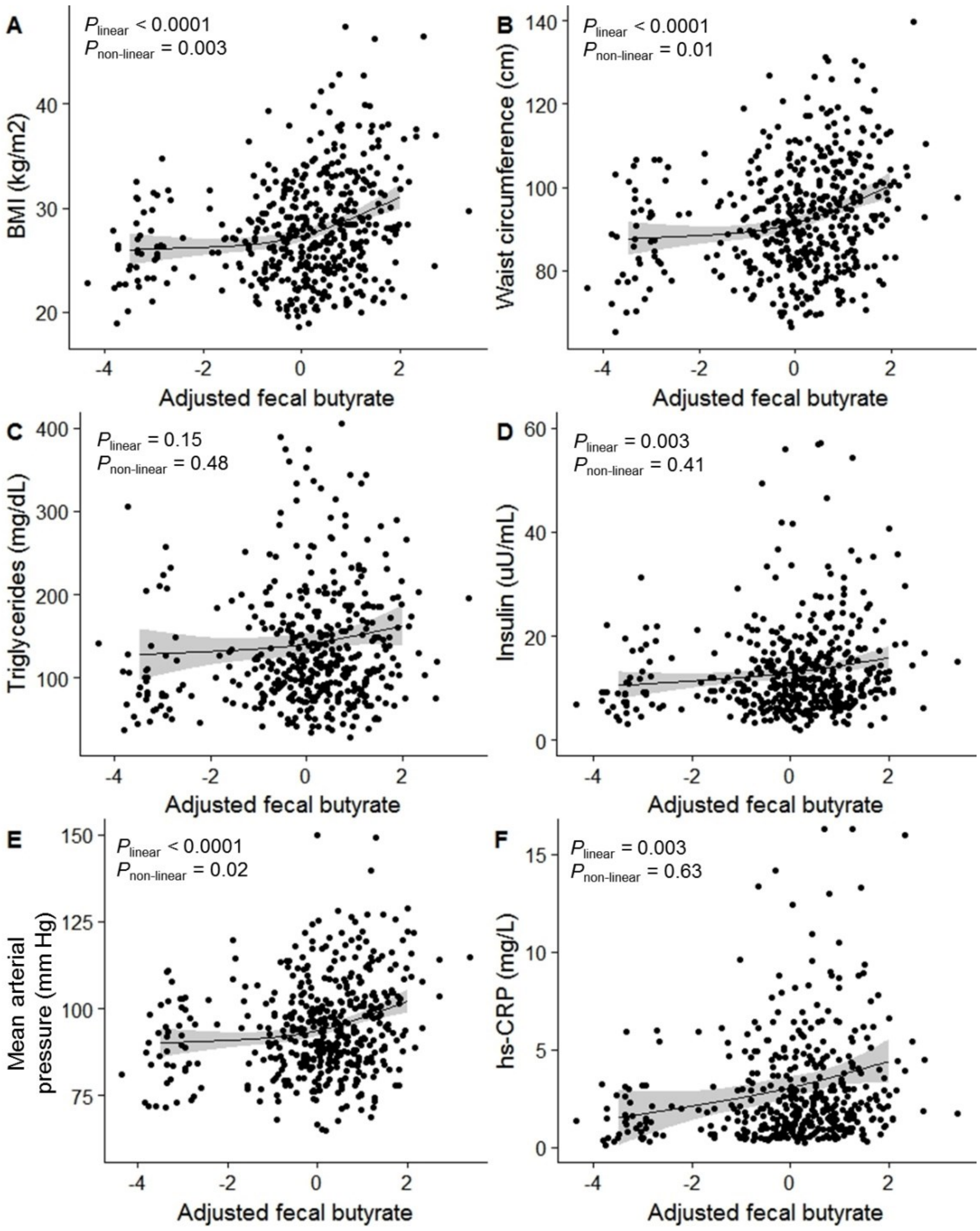


**Figure S2.** Distribution of cardiometabolic health indicators according to multivariable-adjusted fecal butyrate concentration. Butyrate concentrations adjusted for age, city of origin, caloric intake, physical activity and fiber intake. Restricted cubic splines fits with 95% confidence intervals are shown. P-values from ordinary least squares testing the linearity and non-linearity of the regression are also shown. BMI: body mass index; hs-CRP: high-sensitivity C-reactive protein.

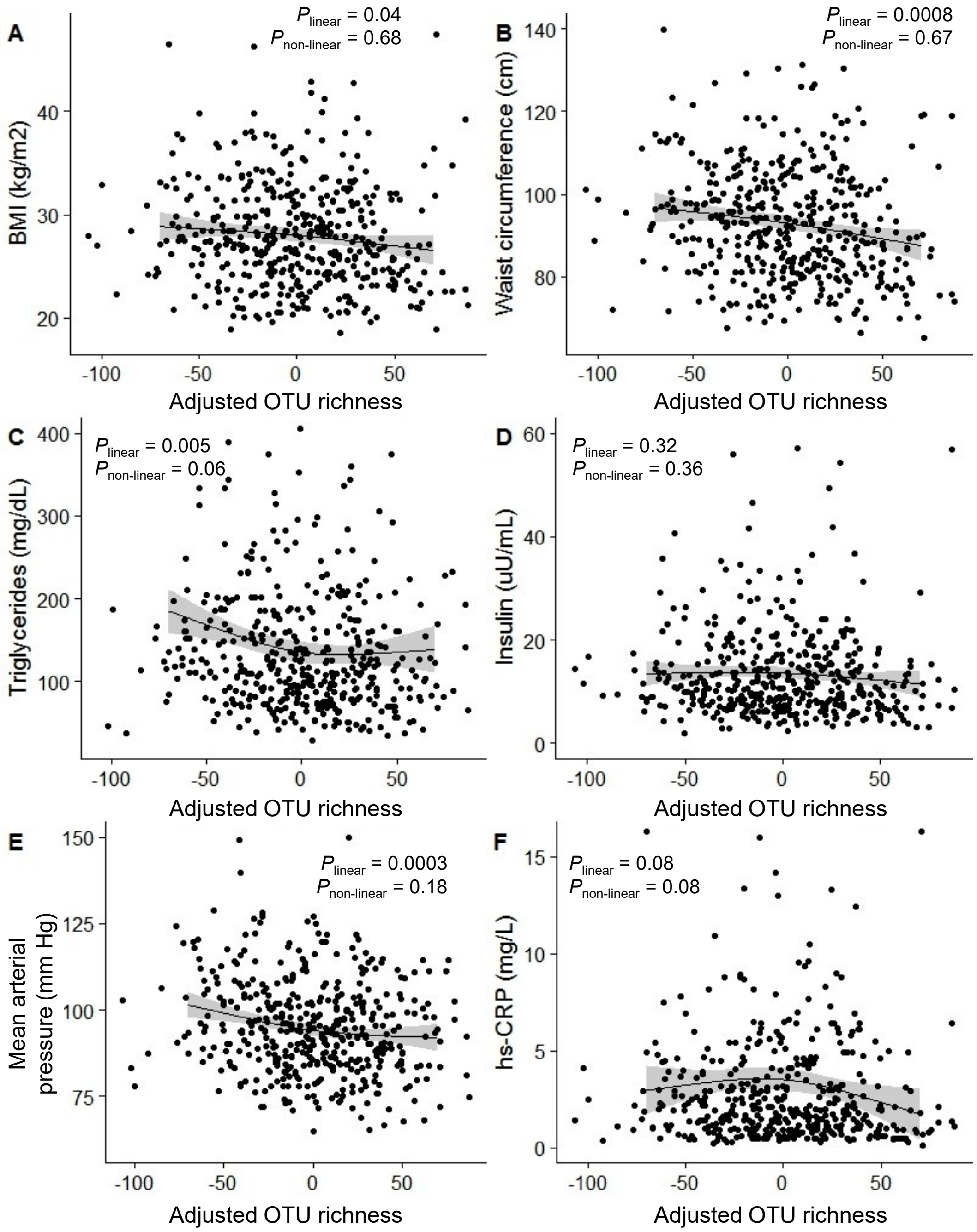


**Figure S3.** Distribution of cardiometabolic health indicators according to multivariable-adjusted gut microbiota diversity. OTU richness adjusted for age, city of origin, caloric intake, physical activity and fiber intake. Restricted cubic splines fits with 95% confidence intervals are shown. P-values from ordinary least squares testing the linearity and non-linearity of the regression are also shown. BMI: body mass index; hs-CRP: high-sensitivity C-reactive protein.

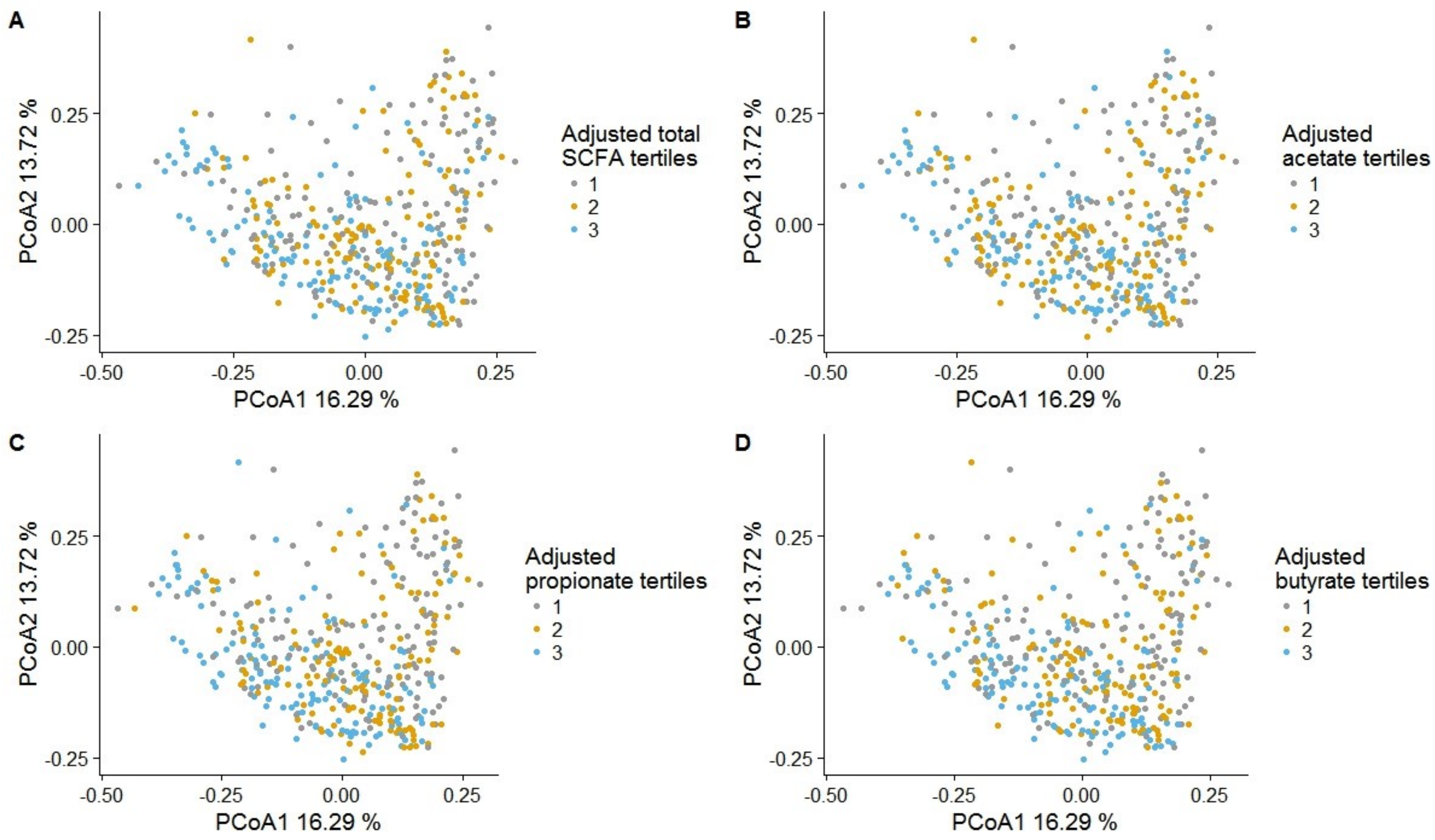


**Figure S4.** Principal coordinates analysis (PCoA) plot based on weighted UniFrac distances. Samples colored by tertiles (1: low, 2: intermediate, 3: high) of multivariable-adjusted fecal SCFA concentrations. (**A**) Total SCFAs, (**B**) acetate, (**C**) propionate, (**D**) butyrate. Percentages on the axes represent the proportion of the explained variation of each component of the PCoA.

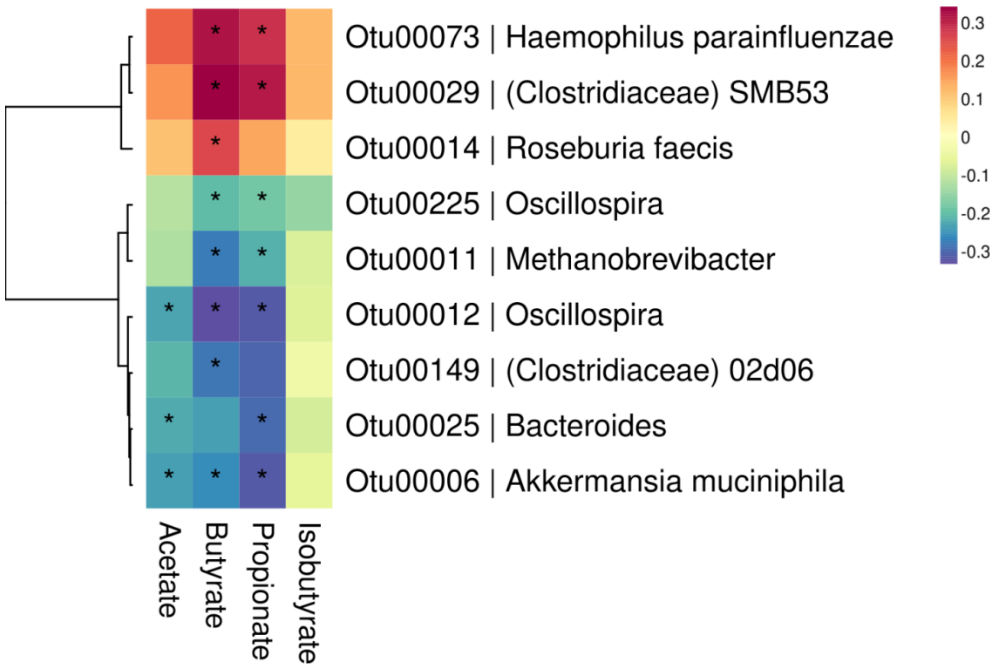


**Figure S5.** Heatmap showing the correlations between rarefied OTU abundances and multivariable-adjusted fecal SCFA concentrations in the subset of participants who did not report smoking or use of pharmacological treatments (N=217). OTUs with moderate or strong association with at least one of the measured SCFAs are shown (|rho|>0.2). The dendrogram to the left was obtained by hierarchical Ward-linkage clustering based on correlation coefficients of the relative abundances of the OTUs that had median abundances ≥0.001%. Models adjusted for age, city of origin, caloric intake, physical activity and fiber intake. The color scale indicates the Spearman's correlation coefficients. FDR-adjusted p-values from quasi-Poisson generalized linear models are indicated (*=$q$<0.10).
